# Role of the Metabolic Profile in Mediating the Relationship Between Body Mass Index and Left Ventricular Mass in Adolescents: Analysis of a Prospective Cohort Study

**DOI:** 10.1161/JAHA.120.016564

**Published:** 2020-10-08

**Authors:** Alice R. Carter, Diana L. Santos Ferreira, Amy E. Taylor, Deborah A. Lawlor, George Davey Smith, Naveed Sattar, Nishi Chaturvedi, Alun D. Hughes, Laura D. Howe

**Affiliations:** ^1^ MRC Integrative Epidemiology Unit Population Health Sciences University of Bristol United Kingdom; ^2^ National Institute for Health Research Biomedical Research Centre at the University Hospitals Bristol NHS Foundation Trust and the University of Bristol Bristol United Kingdom; ^3^ Institute of Cardiovascular and Medical Science University of Glasgow United Kingdom; ^4^ Institute of Cardiovascular Science University College London London United Kingdom

**Keywords:** adiposity, ALSPAC, cardiac structure, mediation, metabolic profile, Risk Factors, Epidemiology, Cardiovascular Disease, Obesity

## Abstract

**Background:**

We aimed to quantify the role of the plasma metabolic profile in explaining the effect of adiposity on cardiac structure.

**Methods and Results:**

Body mass index (BMI) was measured at age 11 in the Avon Longitudinal Study of Parents and Children. Left ventricular mass indexed to height^2.7^ (LVMI) was assessed by echocardiography at age 17. The metabolic profile was quantified via ^1^H‐nuclear magnetic resonance spectroscopy at age 15. Multivariable confounder (maternal age, parity, highest qualification, maternal smoking, prepregnancy BMI, prepregnancy height, household social class, adolescent birthweight, adolescent smoking, fruit and vegetable consumption, and physical activity)–adjusted linear regression estimated the association of BMI with LVMI and mediation by metabolic traits. We considered 156 metabolomic traits individually and jointly as principal components explaining 95% of the variance in the nuclear magnetic resonance platform and assessed whether the principal components for the metabolic traits added to the proportion of the association explained by putative cardiovascular risk factors (systolic and diastolic blood pressures, insulin, triglycerides, low‐density lipoprotein cholesterol, and glucose). A 1 kg/m^2^ higher BMI was associated with a 0.70 g/m^2.7^ (95% CI, 0.53–0.88 g/m^2.7^) and 0.66 g/m^2.7^ (95% CI, 0.53–0.79 g/m^2.7^) higher LVMI in males (n=437) and females (n=536), respectively. Putative risk factors explained 3% (95% CI, 2%–5%) of this association in males, increasing to 10% (95% CI, 8%–13%) when including metabolic principal components. In females, the standard risk factors explained 3% (95% CI, 2%–5%) of the association and did not increase when including the metabolic principal components.

**Conclusions:**

The addition of the nuclear magnetic resonance‐measured metabolic traits appears to mediate more of the association of BMI on LVMI than the putative risk factors alone in adolescent males, but not females.

**Nonstandard Abbreviations and Acronyms**
ALSPAC: Avon Longitudinal Study of Parents and Children
DBP: diastolic blood pressure
DXA: dual x‐ray absorptiometry
LAI: left arterial index
LVIDD: left ventricular internal diameter
LVMI: left ventricular mass indexed to height2.7
NMR: nuclear magnetic resonance
PC: principal components
RWT: relative wall thickness
SBP: systolic blood pressure

**Clinical Perspective**

**What Is New?**

- A number of cardiovascular risk factors have been identified as putative mediators between body mass index and cardiac structure, including systolic and diastolic blood pressures, insulin, triglycerides, low‐density lipoprotein cholesterol, and glucose. However, much of the effect remains unexplained.
- In an adolescent cohort, the nuclear magnetic resonance–measured metabolic profile appeared to mediate more of the association between body mass index and left ventricular mass indexed to height^2.7^ than putative risk factors alone in adolescent males, but not females.
- There was little evidence that any individual metabolic trait mediated the association between body mass index and left ventricular mass indexed to height^2.7^, in both males and females.

**What Are the Clinical Implications?**

- The metabolic profile may present additional targets for lifestyle or pharmaceutical interventions to reduce the harmful effect of adiposity on cardiovascular health, particularly in males.
- To have large effects, interventions would require broad approaches to improve whole lipid or lipoprotein profiles and some other small molecules, rather than targeting individual measures.

Cardiovascular disease (CVD) remains the leading cause of death globally,^1^ and adiposity is a key CVD risk factor.^2^ Mediation analysis can be used to gain a wider etiologic understanding of an exposure, in addition to identifying modifiable intermediate variables linking the exposure to a particular outcome.^3^ Interventions to prevent or treat high levels of adiposity have had limited impact; therefore, identifying novel modifiable intermediate processes between adiposity and CVD provide an opportunity for future interventions aiming to reduce risk of CVD.^4^, ^5^, ^6^

Blood pressure, glucose, insulin, and lipid levels have been identified as major contributors to the association between adiposity and CVD. These factors have been estimated to explain 46% and 76% of the association between BMI and coronary heart disease and stroke, respectively.^7^ The availability of metabolomic data in cohort studies, specifically the numerous lipid‐based measures determined via nuclear magnetic resonance (NMR) spectroscopy, has led to an increased understanding of the causal effects of body mass index (BMI) on circulating metabolites^8^ as well as the role of such metabolites on CVD risk.^9^, ^10^ Therefore, metabolic intermediates are strong candidates as intermediates on the causal pathway from adiposity to CVD risk, which importantly, can be intervened on. For example, harmful cholesterol levels are already targeted using statin medication, which is widely prescribed in routine general practice.

Although adverse cardiovascular events largely occur in adult life, cardiovascular pathology has been shown to have its origins in early life,^11^, ^12^, ^13^, ^14^ with levels of adiposity and cardiovascular risk factors known to track from childhood through to adulthood.^15^ Measures of cardiac structure and function in adults are preclinical markers of CVD,^16^ and there is evidence that cardiac structure in young adults is associated with future risk of CVD events.^17^ Previous analyses carried out in the cohort used in this study (ALSPAC [the Avon Longitudinal Study of Parents and Children]) have demonstrated a causal relationship between BMI and left ventricular mass indexed to height^2.7^ (LVMI), a measure of cardiac structure, in adolescents.^18^

In this study, we use data from adolescents in ALSPAC, a UK prospective cohort study, to assess the role of NMR‐measured metabolic traits as mediators of the association between BMI and LVMI. Mediation analysis is inherently a causal inference method, where causality is assumed between the exposure and outcome, exposure and mediator, and mediator and outcome.^3^ Therefore, these analyses focus on the association between BMI and LVMI given the existing evidence for a causal relationship.^18^ Our primary aim is to identify whether considering the whole of the NMR‐measured metabolic profile results in a greater proportion of the BMI–LVMI relationship being explained over and above the amount explained by putative intermediate risk factors (systolic blood pressure [SBP], diastolic blood pressure [DBP], insulin, triglycerides, low‐density lipoprotein cholesterol [LDL‐C], and glucose).

## Methods

### Participants

ALSPAC is a population‐based birth cohort study. Pregnant women living in the former county of Avon, South West England, with an expected delivery date between April 1, 1991 and December 31, 1992, were eligible for enrollment. In total, 14 541 women were enrolled in to ALSPAC, with 14 901 children born. The participants have been followed up since birth, with questionnaires and links with routine data and research clinics. Full details of the cohort have been reported previously.^19^, ^20^ All participants have given informed consent to be involved in the ALSPAC study. Ethical approval for this specific project was obtained from the ALSPAC law and ethics committee and local ethics committees. The study website contains details of all the data that are available through a fully searchable data dictionary and variable search tool (http://www.brist​ol.ac.uk/alspa​c/resea​rcher​s/our-data/).^21^ To maintain temporal sequencing of our exposures, mediators, and outcomes, we used adiposity measures from age 11, metabolic traits assessed at age 15, and cardiac structure assessed at age 17 years.

### Anthropometric Measurements

At the age 11 follow‐up clinic, height was measured using the Harpenden stadiometer, without shoes. Weight was measured using the Tanita body fat analyzer. BMI was then calculated as weight in kilograms divided by the square of height in meters.

### Mediator Measurements

Fasting (overnight or minimum 6 hours) plasma metabolic traits were quantified via high‐throughput ^1^H‐NMR spectroscopy (referred to as NMR) (Nightingale Health, Helsinki, Finland), at age 15. For samples taken in the morning the fasting period was overnight and for afternoon samples (after 14:00) individuals were required to fast for at least 6 hours. The protocol for this method and uses of this method in epidemiologic analyses has been described extensively in the literature.^22^, ^23^, ^24^ In brief, NMR spectroscopy detects all signatures from all components containing protons. Three main molecular windows are identified; (1) the LIPO window, which characterizes macromolecules, mainly those of lipoprotein lipids; (2) the low‐molecular‐weight molecule, which suppresses macromolecules and identifies smaller solutes such as amino acids and glycolysis‐related metabolites; and (3) LIPID, which identifies serum lipid constituents.^9^ Traits are mostly quantified in clinically meaningful concentrations (eg, mmol/L). Fatty acids are considered in original units and as ratios to total fatty acids. A total of 229 metabolic traits were measured, consisting of 149 concentration measures and 80 ratio measures. With the exception of fatty acid ratios, all other ratios measured were excluding resulting in 156 metabolites for analysis. These 156 metabolic traits represent 14 lipoprotein subclasses and covering a broad spectrum of metabolic pathways (Table S1).

Putative mediators were identified from the literature, where there was existing causal evidence of them being (1) affected by adiposity or anthropometric traits and (2) independent risk factors for CVD or they had previously been identified as mediators of the association. Metabolic traits included as putative mediators were measured using fasting plasma glucose samples. Fasting plasma glucose was measured using an automated assay. Insulin was measured from blood samples using an enzyme‐linked immunosorbent assay (Mercodia, Uppsala, Sweden). Plasma lipid concentrations, including triglycerides and LDL‐C, were taken from venous blood samples and measured by using enzymatic reagents for lipid determination. The Friedewald equation was used to estimate LDL‐C.^25^ Where traits, such as LDL‐C are measured both in the NMR platform and as putative mediators from plasma glucose, the traits were excluded from the NMR platform (see Statistical Analysis full details).

Resting SBP and DBP were measured at least twice during clinics, using a Dinamap 9301 vital signs monitor (Morton Medical, London, UK) and cuff size appropriate for the child. A mean of the final two measures was used.

### Cardiac Structure Measures

Left ventricular mass was assessed by echocardiography in a quasi‐random subset of participants in ALSPAC at the age 17 clinic. Echocardiography was performed using a HDI 5000 ultrasound machine (Philips) equipped with a P4‐2 phased‐array ultrasound transducer. All measurements were made according to the American Society of Echocardiography guidelines, and validated equations were used to calculate LVMI.^26^

### Confounder Assessment

**Figure 1:**
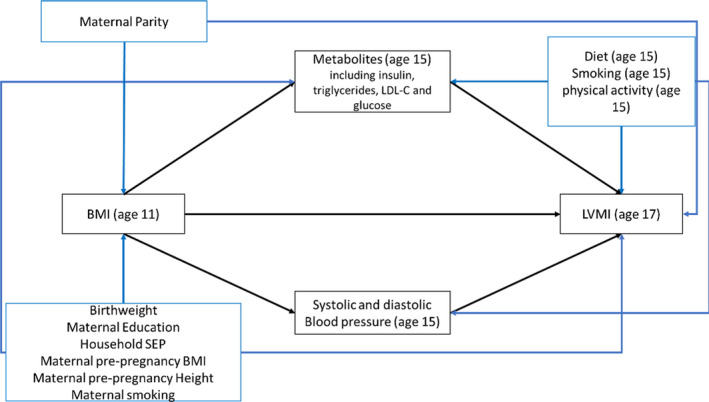
Directed acyclic graph depicting causal assumptions made in mediation analyses assessing the role of metabolic mediators on the association between BMI and LVMI. BMI indicates body mass index; LDL‐C, low‐density lipoprotein cholesterol; LVMI, left ventricular mass indexed to height^2.7^; and SEP, socioeconomic position.

Mediation assumes causal effects and therefore that there is no confounding between the exposure and outcome, exposure and mediator, and mediator and outcome as well as no intermediate confounders (that being a confounder of the mediator and outcome that is itself influenced by the exposure).^3^ Confounders included in analyses were selected based on a priori knowledge and were included in all analysis models as either confounders of the exposure and mediator, mediator and outcome, or exposure and outcome or between all three (see Figure 1). Maternal confounders in this analysis were age, parity, education, prepregnancy height, prepregnancy BMI, and smoking. Adolescent confounders were birthweight, smoking (at age 15), physical activity (at age 15), and diet (at age 15) measured by fruit and vegetable intake. Household social class, around the time of pregnancy, was also included as a confounder. Full details of all confounders and their measurement are provided in Data S1.

Participants were excluded if a value below zero was recorded for any anthropometric trait (BMI, waist circumference and dual X‐ray absorptiometry (DXA)‐determined fat mass; n=22 excluded). Additionally, one individual was excluded because he or she was an analytical outlier on the NMR platform. The Friedewald equation used to measure LDL‐C excludes samples with a plasma triglyceride level of >400 mg/dL; no individuals included in this analysis met this criterion and no exclusions were made. Confounders with a value below zero (mainly reflecting missing data) were recoded as missing and multiply imputed as with other missing data (Table S2).

### Statistical Analysis

All analyses were run on Stata 15; statistical code is available from the corresponding author on request. Access to the ALSPAC data resource can be requested through the executive committee. Based on previous literature indicating different cardiac risk profiles in males and females, it was decided a priori to carry out all analyses stratified by sex.^27^, ^28^, ^29^, ^30^

Multivariable linear regression was used to test the association between (1) BMI and LVMI (total effect), (2) the association between BMI and each metabolic trait individually, and (3) the association between each individual metabolic trait and LVMI. All analyses were adjusted for the confounders specified in the previous section.

Because mediation analysis assumes causal effects it uses a terminology (eg, total effects) to reflect that, as we do here (we discuss the extent to which the assumptions of mediation analyses are likely to be violated under Discussion). Several mediation models were carried out to assess the extent to which the total effect was explained by the metabolomic profile and putative risk factors. The models considered were (1) each metabolic trait considered individually; (2) all traits in the NMR‐measured metabolic platform considered together (as principal components [PCs]); (3) a set of putative cardiovascular risk factors (SBP, DBP, insulin, triglycerides, LDL‐C, and glucose); and (4) the putative cardiovascular risk factors and NMR‐measured metabolic traits (as PCs, described below) together. When considered individually, the NMR‐measured metabolic traits were standardized to set the means to 0 and SDs to 1.

Mediation was assessed in a counterfactual framework, where interactions between BMI and NMR‐measured metabolic traits were allowed in individual mediation models, and in multiple mediator models we assumed no interaction between BMI and the mediators.^31^ We report natural direct effects (the effect of BMI on LVMI not via mediators, for a 1 kg/m^2^ increase in BMI where the value of the mediator is allowed to vary for each individual) and natural indirect effects (the mediated effect of the association between BMI and LVMI, for a 1 SD increase in NMR‐measured metabolic traits).^3^, ^31^, ^32^ The CI for the indirect effect was obtained via bootstrapping with 1000 replications. The proportion mediated is calculated by dividing the indirect effect by the total effect and CIs derived by bootstrapping.

### PCs for Metabolic Traits

In multiple mediator analyses considering multiple NMR‐measured metabolic traits in a single model (models 2–4), PCs of the standardized values of the NMR‐measured metabolic traits were used to account for collinearity. The inclusion of multiple collinear variables in a model can result in inflated standard errors.^33^

Principal component analysis is a data reduction technique, taking a set of correlated variables and extracting a set of uncorrelated PCs. Each PC is a linear combination of the original variables in the data.^34^

A number of putative risk factors (insulin, triglycerides, LDL‐C, and glucose) are included in the NMR‐measured metabolic traits. To avoid double counting these mediators in models considering the role of the NMR‐measured metabolic traits in addition to putative risk factors (model 4), the NMR measurements of these putative risk factors were excluded when generating the PCs.

Principal components were estimated separately for males and females. For use in mediation analysis, we included the number of PCs required to estimate 95% of the variance in the NMR‐measured metabolic traits. For model 2 (all NMR‐measured metabolic traits), this was 18 PCs in the females and 19 PCs in the males. For model 4 (putative risk factors plus NMR‐measured metabolic measures), 20 PCs were included in the analysis of females and 21 PCs for males. Taken together, these PCs capture variation across the metabolic profile. Therefore, we cannot use these analyses to identify the contribution of specific metabolic traits to mediation.

### Multiple Imputation

To maximize power and potentially reduce bias, multivariable multiple imputation was carried out to impute missing confounders. The proportion of missingness is available in Table S2. The sample for imputation was defined as all individuals with complete data on all adiposity variables at ages 11, mediators (including NMR‐measured metabolic platform and putative risk factors) at age 15, and echocardiography data at age 17. The PCs reflecting 95% of the variance in all NMR‐measured metabolic traits were included in the imputation model, rather than all NMR‐measured metabolic traits, to avoid collinearity and convergence problems. We created 20 imputed data sets. The distribution of these imputed variables was assessed to confirm that the imputed data were consistent with the original data. Each imputed data set was analyzed separately, with the results combined using Rubin's rules.

### Sensitivity Analyses

Although sex‐stratified analyses were prespecified a priori, a likelihood ratio test was carried out to test whether a model for the total effect accounting for interaction by sex was a better fit than when interactions were not considered.^27^, ^28^, ^29^ It was determined a priori to use BMI, mediators (including metabolic traits), and LVMI all measured at different time points. The pairwise correlation between BMI measures at age 11 and BMI measured at age 15 was assessed to identify whether BMI was stable across puberty.

In addition to BMI, all analyses (including all individual mediator models and all multiple mediator models) were replicated using waist circumference and DXA‐determined fat mass as measures of adiposity. Three additional measures of cardiac structure that have been linked to cardiovascular health were also considered in sensitivity analyses, namely, left atrial size indexed to height (LAI), left ventricular internal diameter (LVIDD), and relative wall thickness (RWT). In total, the association between each exposure (BMI, waist circumference, and DXA‐determined fat mass) was assessed with each outcome (LVMI, LAI, LVIDD, and RWT). For each of these exposure and outcome combinations the mediating effects of (1) individual metabolic traits, (2) PCs for the metabolic profile, (3) putative risk factors, and (4) putative risk factors plus PCs for the metabolic profile were estimated. Full details of the additional adiposity and cardiac structure measurements are available in Data S1.

From the individual metabolic trait mediation results, small very‐low‐density lipoproteins (VLDL) as a group appeared to have a stronger mediating effect (ie, a larger indirect effect) than other groups of NMR‐measured metabolic traits. Therefore, as a post hoc sensitivity analysis to understand whether the effects of the NMR‐measured metabolic traits considered jointly were driven by the small VLDL class of lipoproteins, we ran sensitivity analyses including only these in a model with putative cardiovascular risk factors across all exposure and outcome combinations.

To evaluate whether total effects and indirect effects were independent of puberty, age at peak height velocity,^35^ an indicator of timing of puberty, was included as a covariate in multiple mediator models assessing mediation between BMI and LVMI with (1) the metabolic PCs and (2) joint model with the metabolic PCs and putative risk factors.

In addition to analyses using imputed data, complete case analyses were carried out for the association between BMI and LVMI and the extent to which the total effect was explained by the mediators considered in the main analyses.

## Results

### Participant Characteristics

**Figure 2:**
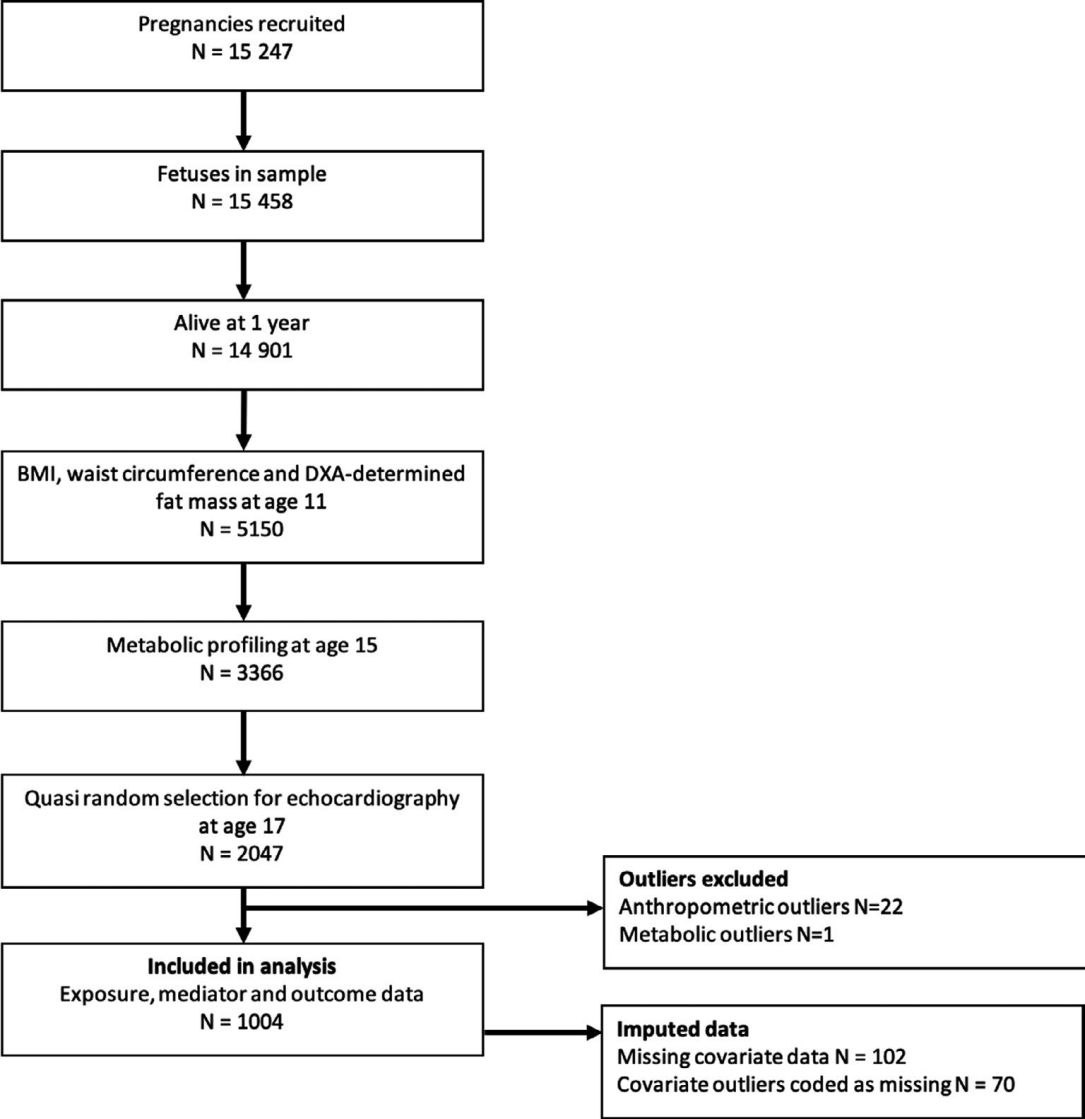
Flow chart of study recruitment to inclusion in analyses. BMI indicates body mass index; and DXA, dual X‐ray absorptiometry.

A total of 1004 participants were eligible for analysis. Of these, 467 were males and 537 were females. A study flow chart is shown in Figure 2. Full participant characteristics are presented in Table 1, and comparisons between the imputed data, nonimputed eligible sample, and whole ALSPAC sample at relevant ages are presented in Table S3.

**Table 1:** Imputed Sample Study Characteristics in All Eligible Participants, Males and Females

	All Participants (N=1004) mean (standard deviation) or proportion (standard error)	Male (n=437) mean (standard deviation) or proportion (standard error)	Female (n=536) mean (standard deviation) or proportion (standard error)
Exposures			
BMI, kg/m^2^	19.07 (3.17)	18.72 (3.00)	19.37 (3.29)
Waist circumference, cm	68.25 (8.85)	68.62 (9.03)	67.93 (8.69)
Total body fat mass, g	15 217.25 (8397.65)	10 889.04 (7246.14)	18 981.27 (7469.77)
Outcomes			
LVMI, g/m^2.7^	28.00 (5.87)	29.92 (5.95)	26.32 (5.27)
LAI	0.00 (0.19)	−0.01 (0.24)	0.00 (0.12)
RWT	0.38 (0.06)	0.39 (0.06)	0.37 (0.06)
LVIDD average, cm	4.53 (0.46)	4.73 (0.49)	4.35 (0.36)
Covariates (offspring)			
Sex (% male)	0.30 (0.03)		
Offspring birthweight, g	3463.90 (525.00)	3549.90 (558.76)	3389.12 (481.91)
Adolescent smoking (% smoked in past 30 d or more)	0.54 (0.03)	0.54 (0.06)	0.54 (0.03)
Frequency of fresh fruit consumption (% consumed less than once per day)	0.83 (0.01)	0.85 (0.02)	0.82 (0.02)
Frequency of fresh vegetable consumption (% consumed less than three times per week)	0.72 (0.01)	0.71 (0.02)	0.72 (0.02)
Physical activity (% takes part in sport with friends)	0.64 (0.02)	0.75 (0.02)	0.55 (0.02)
Covariates (maternal)			
Maternal age	29.50 (4.45)	29.63 (4.30)	29.40 (4.58)
Maternal parity	0.70 (0.83)	0.68 (0.83)	0.72 (0.83)
Maternal prepregnancy BMI	22.95 (3.57)	22.98 (3.43)	22.92 (3.70)
Maternal prepregnancy height (inches)	64.68 (2.68)	64.78 (2.82)	64.60 (2.56)
Maternal smoking (% ever smoker)	0.38 (0.02)	0.36 (0.02)	0.40 (0.02)
Mother's highest qualification			
Less than O‐level	0.15 (0.01)	0.14 (0.02)	0.17 (0.02)
O‐level	0.35 (0.02)	0.34 (0.02)	0.35 (0.02)
A‐level	0.29 (0.01)	0.30 (0.02)	0.27 (0.02)
Degree or above	0.21 (0.01)	0.22 (0.02)	0.20 (0.02)
Household social class			
I (highest)	0.21 (0.01)	0.24 (0.02)	0.19 (0.02)
II	0.45 (0.02)	0.47 (0.02)	0.45 (0.02)
IIINM	0.21 (0.01)	0.18 (0.02)	0.24 (0.02)
IIIM	0.08 (0.01)	0.07 (0.01)	0.08 (0.01)
IV or V (lowest)	0.04 (0.01)	0.04 (0.01)	0.04 (0.01)

BMI indicates body mass index; LAI, left arterial index; LVIDD, left ventricular internal diameter; LVMI, left ventricular mass indexed to height^2.7^; and RWT, relative wall thickness.

### Association Between Adiposity, Risk Factors, and Cardiac Structure

A 1 kg/m^2^ higher BMI in females was associated with an increase in mean LVMI of 0.66 g/m^2.7^ (95% CI, 0.53–0.79 g/m^2.7^). Similarly, in males, a 1 kg/m^2^ higher BMI was associated with an increase in mean LVMI of 0.70 g/m^2.7^ (95% CI, 0.53–0.88 g/m^2.7^; Table 2).

**Table 2:** The Total Effect of BMI on Cardiac Structure Assessed Using Multivariable Linear Regression Stratified by Sex

Exposure (1 kg/m^2^ Increase)	Outcome	Females Mean Difference (95% CI) (n=536)	Males Mean Difference (95% CI) (n=437)
BMI	LVMI	0.661 (0.529 to 0.793)	0.701 (0.525 to 0.877)
	LAI	−0.002 (−0.006 to 0.001)	−0.006 (−0.016 to 0.003)
	LVIDD	0.027 (0.017 to 0.036)	0.012 (0.027 to 0.042)
	RWT	0.001 (−0.0001 to 0.003)	0.002 (2.86 × 10^–5^ to 0.004)

Models adjusted for maternal covariables—age, parity, education, prepregnancy height, prepregnancy BMI, smoking, and household social class; and adolescent covariables—birthweight, smoking, physical activity, and diet. BMI indicates body mass index; LAI, left arterial index; LVIDD, left ventricular internal diameter; LVMI, left ventricular mass indexed to height^2.7^; and RWT, relative wall thickness.

The association between BMI and individual metabolic traits was mixed; for example, BMI was positively associated with all subclasses of VLDL, but there was little evidence of an association between BMI and the low‐density lipoproteins (LDL), fatty acids, or fatty acid ratios. BMI was mostly negatively associated with the high‐density lipoprotein subclass of metabolic traits. There was evidence of a positive association between BMI and branched‐chain amino acids in males, but not in females (Figure S1).

In all VLDL subclasses, there was a positive trend in the association with LVMI in both males and females. With the exception of the triglyceride metabolic traits, large, medium, and small LDL traits were positively associated with LVMI. There was some evidence in males of a positive association between fatty acids and LVMI, although this was less consistent in females. In both males and females, citrate was negatively associated with LVMI. There was some evidence of an association between branched‐chain amino acids and LVMI (Figure S2).

### Mediation of the Association Between Adiposity and Cardiac Structure

**Figure 3:**
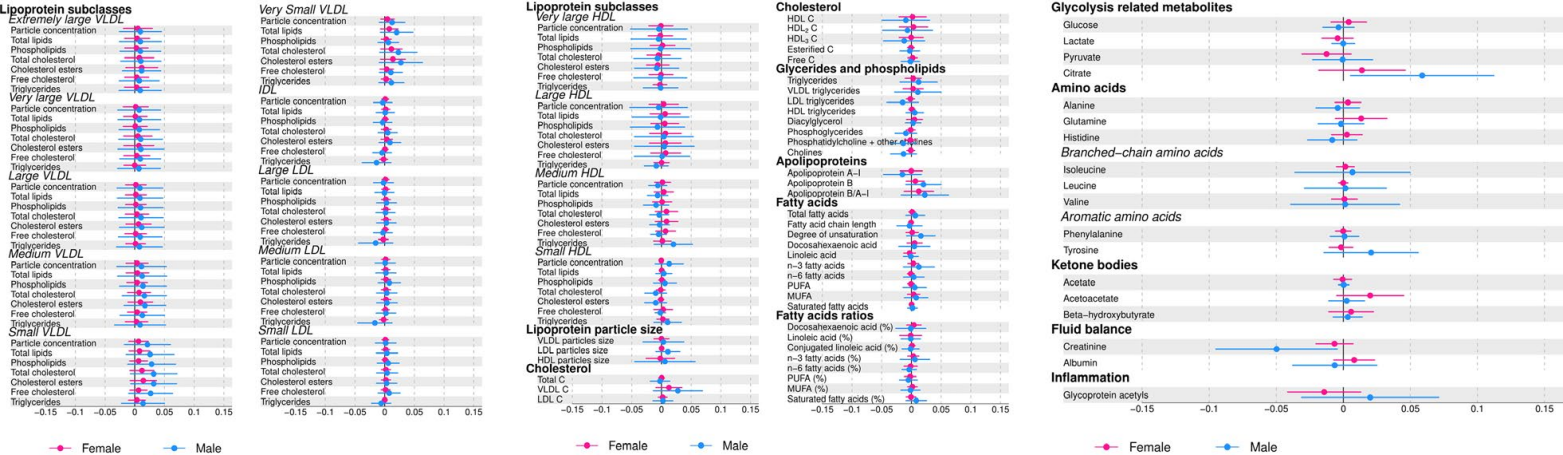
Forest plot showing the natural indirect effect of each NMR‐measured metabolic trait individually on the association between BMI to LVMI stratified by sex. Models adjusted for maternal age, maternal parity, maternal education, maternal prepregnancy height, maternal prepregnancy BMI, maternal smoking, household social class, adolescent birthweight, adolescent smoking, adolescent diet and adolescent physical activity. BMI indicates body mass index; HDL, high‐density lipoprotein; LDL, low‐density lipoprotein; LVMI, left ventricular mass indexed to height; NMR, nuclear magnetic resonance; and VLDL, very‐low‐density lipoprotein. All results are g/m^2.7^ of LVMI per 1 kg/m^2^ higher BMI.

Considered separately, each metabolic trait explained only a small proportion of the association between BMI and LVMI. In males, the median proportion mediated for the association between BMI and LVMI was 0.5% (95% CI, 0.5%–0.5%) and the maximum was 9% (95% CI, 9%–9%; explained by citrate). In females, the median proportion mediated was 0.3% (95% CI, 0.3%–0.3%) and the maximum was 3% (95% CI, 3%–3%; explained by acetoacetate; Figure 3).

Together, the PCs explaining 95% of variance in the NMR‐measured metabolic traits explained 16% (95% CI, 12%–19%) of the association between BMI and LVMI in males, and 5% (95% CI, 3%–6%) in females (Table 3).

**Table 3:** Direct and Indirect Effects of Multiple Mediator Models on the Association Between BMI and LVMI

Mediators	Females (n=536)			Males (n=437)		
	Natural Direct Effect (95% CI)	Natural Indirect Effect (95% CI)	Proportion Mediated % (95% CI)	Natural Direct Effect (95% CI)	Natural Indirect Effect (95% CI)	Proportion Mediated % (95% CI)
Putative risk factors only (SBP, DBP, insulin, triglycerides, LDL‐C, and glucose)	0.64 (0.50, 0.77)	0.02 (−0.01, 0.06)	3.46 (1.91, 5.01)	0.68 (0.50, 0.86)	0.02 (−0.04, 0.09)	3.35 (1.72, 4.99)
NMR‐measured metabolic measures only (as PCs)	0.63 (0.49 to 0.77)	0.03 (−0.03 to 0.09)	4.64 (2.86 to 6.41)	0.59 (0.41 to 0.79)	0.11 (0.003 to 0.22)	15.62 (12.32 to 18.91)
Putative risk factors+NMR‐measured metabolic measures (as PCs)	0.65 (0.51 to 0.78)	0.02 (−0.04 to 0.06)	2.34 (1.06 to 3.62)	0.63 (0.44 to 0.82)	0.07 (−0.02 to 0.17)	10.35 (7.59 to 13.11)
Putative risk factors+small VLDLs	0.63 (0.49 to 0.76)	0.03 (−0.01 to 0.07)	4.81 (3.00 to 6.61)	0.66 (0.48 to 0.85)	0.09 (−0.04 to 0.08)	3.07 (1.51 to 4.64)

Models adjusted for maternal covariables—age, parity, education, prepregnancy height, prepregnancy BMI, smoking, household social class; and adolescent covariables—birthweight, smoking, physical activity, and diet. All results are g/m^2.7^ of LVMI per 1 kg/m^2^ higher BMI. BMI indicates body mass index; DBP, diastolic blood pressure; LDL‐C, low‐density lipoprotein cholesterol; LVMI, left ventricular mass indexed to height^2.7^, NMR, nuclear magnetic resonance; PC, principal components; SBP, systolic blood pressure; and VLDL, very‐low‐density lipoprotein.

The putative cardiovascular risk factors (SBP, DBP, insulin, triglycerides, LDL‐C, and glucose) explained 3% (95% CI, 2%–5%) of the association between BMI and LVMI in males. This increased to 10% (95% CI, 8%–13%) when the metabolic PCs were included in the model alongside the putative risk factors (Table 3).

In females the proportion of the association between BMI and LVMI explained by the putative cardiovascular risk factors was 3% (95% CI, 2%–5%), but when the metabolic PCs were included in the model with the putative mediators this reduced to 2% (95% CI, 1%–4%; Table 3).

### Sensitivity Analyses

There was little evidence of a statistical interaction between males and females for the total effect of BMI on LVMI (*P* value_interaction_=0.51; Table S4). BMI measured at age 11 was highly correlated with BMI measured at age 15 (pairwise correlation=0.8).

The association between waist circumference and separately between DXA‐determined fat mass and individual metabolic traits was consistent with the association between BMI and individual metabolic traits (Figures S3 and S4). There was little evidence of an association between any individual metabolic traits and LAI, LVIDD, and RWT (Figures S5 through S7).

In mediation models considering each metabolic trait individually each metabolic trait explained little of the association between BMI and LAI, LVIDD, or RWT. Similar results were observed for waist circumference and DXA‐determined fat mass with each outcome (Figures S8 through S18).

In multiple mediator analyses, considering BMI as the exposure the metabolic PCs increased the amount explained between BMI and LAI and for the association between BMI and LVIDD, compared with the putative risk factors alone. In females, there was evidence that the metabolic profile mediated more of the effect of BMI on RWT than the putative risk factors alone, but not in males (Table 3).

In multiple mediator models, there was little evidence in females that the PCs for the metabolic traits mediate more of the effect of waist circumference on LVMI compared with the putative risk factors. However, in males, the PCs for the metabolic traits did mediate more of the effect. This pattern of results was similar when considering DXA‐determined fat mass as the exposure. For both waist circumference and DXA‐determined fat mass there was greater evidence of mediation by the metabolic traits when considering LAI and RWT as the outcomes (Figure S19).

In both males and females, the proportion mediated by total small VLDL were higher than for other metabolic subgroups. However, when including the small VLDL with putative the putative mediators they explained no more of the association between BMI and LVMI than the putative mediators alone.

In both males and females, including age at peak height velocity in the models had little effect on the estimates of the proportion mediated (Table S5). The point estimates for the total effects estimated using complete case data were typically larger than those from multiply imputed data, but with wider levels of imprecision (Table S6).

## Discussion

In this cohort of UK adolescents, we have demonstrated in males but not females that the wider metabolic profile may contribute to the burden of CVD attributable to BMI, over and above the amount explained by putative intermediate risk factors alone (SBP, DBP, insulin, triglycerides, LDL‐C, and glucose). Individually, the metabolic traits explained little of the association between BMI and LVMI. These results were consistent when considering additional measures of adiposity (waist circumference and DXA‐determined fat mass) and cardiac structure (LAI, LVIDD, and RWT).

### Results in Context

To our knowledge, no other study has examined the role of NMR‐measured metabolic traits as mediators of the association between BMI and LVMI. With the same data as used in this analysis (ALSPAC), a causal effect of BMI and LVMI has been demonstrated,^18^ providing the motivation for identifying intermediate variables that may mediate this effect. LVMI is a precursor to adverse cardiovascular events in adulthood.^36^ Therefore, identifying intermediate variables from BMI may provide an opportunity to identify potential therapeutic targets.

A recent Mendelian randomization study investigating the mediating effects of lipids and glycemic traits found stronger mediating effects than our results for the putative set of risk factors.^37^ In our analysis we have considered the role of the metabolic profile in adolescence, whereas in a Mendelian randomization analysis, the estimates reflect a lifetime effect of an exposure (or mediator).^36^ Therefore, it may be possible that the mediating role of the metabolic profile between BMI and LVMI (and adiposity and cardiac structure more broadly) emerges throughout the life course.

Sex differences in cardiometabolic profiles have been shown in a number of studies in both children and adults.^28^, ^29^ In a previous study using ALSPAC data, it was shown that the association between BMI and cardiovascular risk factors was stronger in males than females.^28^ Additionally, sex differences in the association of adiposity and the metabolic profile have previously been shown.^8^ Although we found consistent estimates of the proportion mediated by the putative risk factors in males and females for the association between BMI and LVMI, we found some evidence of stronger mediating effects of the NMR‐measured PC profiles in males. Although there was no strong evidence for a statistical difference between males and females, it is likely that we had insufficient statistical power to detect this.

In this analysis, we found less evidence of an association between BMI and individual metabolic traits than previous, larger analyses. Our smaller sample size is likely to be contributing to these differences.^8^ Additionally, previous analyses have used Mendelian randomization to explore the causal effect of BMI on individual metabolic traits, which as previously noted will be estimating lifetime effects of an exposure, which may not yet be present in our adolescent cohort.

Previous studies have found evidence of an association between aromatic amino acids, phenylalanine, and tyrosine and increased CVD risk factors, including insulin, SBP, and DBP,^38^ in addition to incident cardiovascular events.^39^ However, in this analysis, we only found evidence of an association between tyrosine and LVMI in males.

We also found evidence of an association between BMI and branched‐chain amino acids in males, but not females. Additionally, in both males and female, branched‐chain amino acids were positively associated with LVMI. However, there was little evidence that they mediated the association of BMI and LVMI. Associations have previously been identified between branched‐chain amino acids and diabetes^40^ and CVD.^41^, ^42^ These previous studies have been in adult populations; therefore, these effects may not yet be present in our adolescent population.

### Strengths and Limitations

In this multivariable regression analysis, residual confounding of associations cannot be ruled out. We controlled for all measured potential confounders of the exposure and outcome, exposure and mediator, and mediator and outcome associations, but residual confounding may be present where the variables included in analyses fail to accurately measure the confounder. For example, diet was considered a confounder, and we adjusted for fruit and vegetable intake. However, the confounding effect of diet between BMI and LVMI is likely to be more complex than just considering fruit and vegetable intake. Adolescent smoking was considered as a confounder of the mediator (including metabolic traits and blood pressure traits) and LVMI association in this analysis. However, there is evidence of bidirectional associations between BMI and smoking, where although increased smoking is widely reported to lead to reduced BMI,^43^ there is some evidence that increased BMI is associated with increased smoking,^44^ and smoking could also be a mediator of BMI and LVMI. As such, there is potential for overadjustment by including smoking in the model. However, in this adolescent cohort we expect the strongest relationship is likely to be smoking influencing the mediators and therefore we adjusted for smoking.

Mediation analysis could be biased by reverse causality due to a misspecified model, for example, if the metabolic profile influenced adiposity rather than the converse. All variables considered were measured prospectively, with appropriate temporal ordering of the exposure, mediators, and outcomes, alleviating concerns over reverse causality or bias from the use of cross‐sectional data in mediation analysis.^45^ Additionally, as an adolescent population, individuals included in these analyses are unlikely to have experienced an adverse major cardiac event or be on preventative medication for cardiovascular diseases (such as statins). This further lessens concerns over reverse causality and potential bias caused by treatment effects.

It is possible that age 11 is too young to clearly identify the effects of BMI on metabolites and subsequently LVMI, particularly as trajectories of BMI are shown to change through puberty.^28^ However, given the high correlation between BMI at age 11 and BMI at age 15 in this cohort where the pairwise correlation for BMI at both ages was 0.8, the results presented here are unlikely to be biased by trajectories of BMI during puberty.

In addition to reverse causality and residual confounding, mediation analysis can be biased by measurement error, particularly in the mediator.^46^ This analysis uses objectively measured metabolic data, representing a broad range of metabolic traits, typically not captured by standard biochemical assays. However, these measures are only a snapshot of one time point (age 15) and may not be capturing the full life course effect of these metabolic traits.

Although the primary analyses focused on the association between BMI and LVMI, other measures of adiposity and cardiac structure were considered in analyses. BMI is often criticized as a poor indicator of overall adiposity, particularly due to its inability to differentiate between lean and fat mass. DXA‐determined fat mass may be a better measure for distinguishing between types of body fat and assessing overall adiposity.^47^ However, consistent with previous analyses in ALSPAC^28^ and other cohorts,^48^ our estimates of mediation were similar when waist circumference of DXA‐determined fat mass were considered as exposures instead of BMI.

The ALSPAC sample is a large contemporary cohort with more than 14 000 participants enrolled in the original cohort. However, when the analysis was restricted to the subset of individuals with all relevant data on anthropometry, NMR‐measured metabolic platform, putative cardiovascular risk factors, and cardiac structure the sample was just over 1000 individuals. Our findings need to be replicated in a larger cohort, particularly if replication could involve using causal inference methods such as Mendelian randomization to triangulate results.^49^, ^50^ However, instrumenting the multiple metabolic traits may prove challenging.

A limitation of examining these mediating effects in a younger cohort is that some effects of either the exposure or the metabolic profile may only become apparent later in life. As more large‐scale biobanks with adult populations release metabolic data, replicating these analyses in adult populations would be important to see whether these results are replicated with clinical CVD events as outcomes.

### Clinical and Public Health Implications

We show that metabolic traits, acting together, mediate some of the effect of BMI on cardiac structure in adolescence. In these analyses, we have not identified a clear intervenable target by a single lipid or metabolic trait or metabolic group. The PCs included in mediation analysis reflect the variation in metabolic traits across the metabolic profile. To this extent, they are unlikely to be estimating the effect of a single metabolic trait or metabolic group. Rather, they explore the effect across the metabolic profile. Early intervention on these multiple mediators might therefore be a useful strategy to reduce future cardiovascular disease. Future studies examining the effect of interventions such as exercise or dietary modification on complex metabolic profiles may be useful in guiding CVD prevention strategies in young people.

## Conclusions

This study demonstrates that in an adolescent population, the metabolic profile may present additional targets for lifestyle or pharmaceutical interventions to reduce the harmful effect of adiposity on cardiovascular health, particularly in males. However, our results suggest that to have large effects, interventions would require broad approaches to improve whole lipid or lipoprotein profiles and some other small molecules, rather than targeting individual measures. Furthermore, these findings need replication in larger independent samples, analyses to establish causality, and to be explored in adult populations to investigate whether this association is observed with clinical CVD outcomes.

## Sources of Funding

A. Carter, Dr Santos Ferreira, Dr Taylor, Dr Lawlor, Dr Davey Smith, and Dr Howe all work in a unit supported by the UK Medical Research Council and the University of Bristol (Program codes: MC_UU_00011/1 and MC_UU_00011/6]. A. Carter is supported by a UK Medical Research Council PhD Studentship (MC_UU_00011/1). Dr Lawlor, Dr Taylor, and Dr Davey Smith are supported by the National Institute for Health Research (NIHR) Biomedical Research Centre based at University Hospitals Bristol NHS Foundation and the University of Bristol. The views expressed are those of the authors and not necessarily those of the NHS, the NIHR, or the Department of Health. Dr Lawlor's contribution is supported by the European Union's Horizon 2020 Research and Innovation Programme Grant 733206 (LifeCycle). The UK Medical Research Council and Wellcome (Grant 217065/Z/19/Z) and the University of Bristol provide core support for ALSPAC. NMR metabolomics data was funded by the MRC (Grant MC_UU_12013/1) AH received support from the Wellcome Trust (086676/7/08/Z) and the British Heart Foundation (PG/06/145 & CS/15/6/31468) for cardiovascular measures in ALSPAC and works in a unit that receives support from the UK Medical Research Council (Program Code MC_UU_12019/1). Howe is supported by a Career Development Award fellowship from the UK Medical Research Council (MR/M020894/1). No funding body has influenced data collection, analysis, or its interpretations. This publication is the work of the authors, who serve as the guarantors for the contents of this paper.

## Disclosures

Dr Lawlor has received support from several national and international government and charitable funders, as well as Medtronic Ltd and Roche Diagnostics in the past 10 ‐years, for work unrelated to that presented here. The remaining authors have no disclosures to report.

## Supporting information


**Data S1**
**Tables S1–S6**
**Figures S1–S19**

## References

[1] Collaborators GBDCoD . Global, regional, and national age‐sex specific mortality for 264 causes of death, 1980–2016: a systematic analysis for the Global Burden of Disease Study 2016. Lancet. 2017;1151–1210. 10.1016/S0140-6736(17)32152-9 PMC5605883 28919116

[2] Dale CE , Fatemifar G , Palmer TM , White J , Prieto‐Merino D , Zabaneh D , Engmann JEL , Shah T , Wong A , Warren HR , et al. Causal associations of adiposity and body fat distribution with coronary heart disease, stroke subtypes, and type 2 diabetes mellitus: a Mendelian randomization analysis. Circulation. 2017;2373–2388. 10.1161/CIRCULATIONAHA.116.026560 PMC5515354 28500271

[3] VanderWeele TJ . Mediation analysis: a practitioner's guide. Annu Rev Public Health. 2016;17–32. 26653405 10.1146/annurev-publhealth-032315-021402

[4] Hardeman W , Griffin S , Johnston M , Kinmonth AL , Wareham NJ . Interventions to prevent weight gain: a systematic review of psychological models and behaviour change methods. Int J Obes Relat Metab Disord. 2000;131–143. 10702762 10.1038/sj.ijo.0801100

[5] Crawford D . Population strategies to prevent obesity. BMJ. 2002;728–729. 12364289 10.1136/bmj.325.7367.728 PMC1124263

[6] Lombard CB , Deeks AA , Teede HJ . A systematic review of interventions aimed at the prevention of weight gain in adults. Public Health Nutr. 2009;2236–2246. 19650959 10.1017/S1368980009990577

[7] Global Burden of Metabolic Risk Factors for Chronic Diseases C , Lu Y , Hajifathalian K , Ezzati M , Woodward M , Rimm EB , Danaei G . Metabolic mediators of the effects of body‐mass index, overweight, and obesity on coronary heart disease and stroke: a pooled analysis of 97 prospective cohorts with 1.8 million participants. Lancet. 2014;970–983. 24269108 10.1016/S0140-6736(13)61836-X PMC3959199

[8] Wurtz P , Wang Q , Kangas AJ , Richmond RC , Skarp J , Tiainen M , Tynkkynen T , Soininen P , Havulinna AS , Kaakinen M , et al. Metabolic signatures of adiposity in young adults: Mendelian randomization analysis and effects of weight change. PLoS Med. 2014;9:e1001765. 10.1371/journal.pmed.1001765 PMC4260795 25490400

[9] Fischer K , Kettunen J , Wurtz P , Haller T , Havulinna AS , Kangas AJ , Soininen P , Esko T , Tammesoo ML , Magi R , et al. Biomarker profiling by nuclear magnetic resonance spectroscopy for the prediction of all‐cause mortality: an observational study of 17,345 persons. PLoS Med. 2014;9:e1001606. 10.1371/journal.pmed.1001606 PMC3934819 24586121

[10] Vaarhorst AA , Verhoeven A , Weller CM , Bohringer S , Goraler S , Meissner A , Deelder AM , Henneman P , Gorgels AP , van den Brandt PA , et al. A metabolomic profile is associated with the risk of incident coronary heart disease. Am Heart J. 2014;45–52.e7. 10.1016/j.ahj.2014.01.019 24952859

[11] Halcox JP , Deanfield JE . Childhood origins of endothelial dysfunction. Heart. 2005;1272–1274. 16162614 10.1136/hrt.2005.061317 PMC1769127

[12] McGill HC Jr , McMahan CA , Herderick EE , Malcom GT , Tracy RE , Strong JP . Origin of atherosclerosis in childhood and adolescence. Am J Clin Nutr. 2000;1307S–1315S. 11063473 10.1093/ajcn/72.5.1307s

[13] Baker JL , Olsen LW , Sorensen TI . Childhood body‐mass index and the risk of coronary heart disease in adulthood. N Engl J Med. 2007;2329–2337. 10.1056/NEJMoa072515 PMC3062903 18057335

[14] McCarron P , Smith GD , Okasha M , McEwen J . Blood pressure in young adulthood and mortality from cardiovascular disease. Lancet. 2000;1430–1431. 10791531 10.1016/S0140-6736(00)02146-2

[15] de Swiet M , Fayers P , Shinebourne EA . Blood pressure in first 10 years of life: the Brompton study. BMJ. 1992;23–26. 10.1136/bmj.304.6818.23 PMC1880926 1734987

[16] Krumholz HM , Larson M , Levy D . Prognosis of left ventricular geometric patterns in the Framingham Heart Study. J Am Coll Cardiol. 1995;879–884. 10.1016/0735-1097(94)00473-4 7884091

[17] Armstrong AC , Liu K , Lewis CE , Sidney S , Colangelo LA , Kishi S , Ambale‐Venkatesh B , Arynchyn A , Jacobs DR Jr , Correia LC , et al. Left atrial dimension and traditional cardiovascular risk factors predict 20‐year clinical cardiovascular events in young healthy adults: the CARDIA study. Eur Heart J Cardiovasc Imaging. 2014;893–899. 24534011 10.1093/ehjci/jeu018 PMC4215562

[18] Wade KH , Chiesa ST , Hughes AD , Chaturvedi N , Charakida M , Rapala A , Muthurangu V , Khan T , Finer N , Sattar N . Assessing the causal role of body mass index on cardiovascular health in young adults: Mendelian randomization and recall‐by-genotype analyses. Circulation. 2018;2187–2201. 30524135 10.1161/CIRCULATIONAHA.117.033278 PMC6250296

[19] Boyd A , Golding J , Macleod J , Lawlor DA , Fraser A , Henderson J , Molloy L , Ness A , Ring S , Davey SG . Cohort profile: the 'children of the 90s'—the index offspring of the Avon Longitudinal Study of Parents and Children. Int J Epidemiol. 2013;111–127. 22507743 10.1093/ije/dys064 PMC3600618

[20] Fraser A , Macdonald‐Wallis C , Tilling K , Boyd A , Golding J , Davey Smith G , Henderson J , Macleod J , Molloy L , Ness A , et al. Cohort profile: the Avon Longitudinal Study of Parents and Children: ALSPAC mothers cohort. Int J Epidemiol. 2013;97–110. 22507742 10.1093/ije/dys066 PMC3600619

[21] Avon longitudinal study of parents and children. Explore data and samples. Available at: http://www.brist​ol.ac.uk/alspa​c/resea​rcher​s/our-data/. Accessed September 3, 2018.

[22] Soininen P , Kangas AJ , Wurtz P , Tukiainen T , Tynkkynen T , Laatikainen R , Jarvelin MR , Kahonen M , Lehtimaki T , Viikari J , et al. High‐throughput serum NMR metabonomics for cost‐effective holistic studies on systemic metabolism. Analyst. 2009;1781–1785. 19684899 10.1039/b910205a

[23] Soininen P , Kangas AJ , Wurtz P , Suna T , Ala‐Korpela M . Quantitative serum nuclear magnetic resonance metabolomics in cardiovascular epidemiology and genetics. Circ Cardiovasc Genet. 2015;192–206. 25691689 10.1161/CIRCGENETICS.114.000216

[24] Inouye M , Kettunen J , Soininen P , Silander K , Ripatti S , Kumpula LS , Hamalainen E , Jousilahti P , Kangas AJ , Mannisto S , et al. Metabonomic, transcriptomic, and genomic variation of a population cohort. Mol Syst Biol. 2010;441. 21179014 10.1038/msb.2010.93 PMC3018170

[25] Friedewald WT , Levy RI , Fredrickson DS . Estimation of the concentration of low‐density lipoprotein cholesterol in plasma, without use of the preparative ultracentrifuge. Clin Chem. 1972;499–502. 4337382

[26] Lang RM , Bierig M , Devereux RB , Flachskampf FA , Foster E , Pellikka PA , Picard MH , Roman MJ , Seward J , Shanewise JS , et al. Recommendations for chamber quantification: a report from the American Society of Echocardiography's Guidelines and Standards Committee and the Chamber Quantification Writing Group, developed in conjunction with the European Association of Echocardiography, a branch of the European Society of Cardiology. J Am Soc Echocardiogr. 2005;1440–1463. 16376782 10.1016/j.echo.2005.10.005

[27] Peters SA , Singhateh Y , Mackay D , Huxley RR , Woodward M . Total cholesterol as a risk factor for coronary heart disease and stroke in women compared with men: a systematic review and meta‐analysis. Atherosclerosis. 2016;123–131. 10.1016/j.atherosclerosis.2016.03.016 27016614

[28] Lawlor DA , Benfield L , Logue J , Tilling K , Howe LD , Fraser A , Cherry L , Watt P , Ness AR , Davey Smith G , et al. Association between general and central adiposity in childhood, and change in these, with cardiovascular risk factors in adolescence: prospective cohort study. BMJ. 2010;c6224. 21109577 10.1136/bmj.c6224 PMC2992109

[29] Lew J , Sanghavi M , Ayers CR , McGuire DK , Omland T , Atzler D , Gore MO , Neeland I , Berry JD , Khera A , et al. Sex‐based differences in cardiometabolic biomarkers. Circulation. 2017;544–555. 28153991 10.1161/CIRCULATIONAHA.116.023005 PMC5302552

[30] Bell JA , Carslake D , O'Keeffe LM , Frysz M , Howe LD , Hamer M , Wade KH , Timpson NJ , Davey SG . Associations of body mass and fat indexes with cardiometabolic traits. J Am Coll Cardiol. 2018;3142–3154. 30545453 10.1016/j.jacc.2018.09.066 PMC6290112

[31] Valeri L , Vanderweele TJ . Mediation analysis allowing for exposure‐mediator interactions and causal interpretation: theoretical assumptions and implementation with SAS and SPSS macros. Psychol Methods. 2013;137–150. 23379553 10.1037/a0031034 PMC3659198

[32] Vanderweele TJ . Controlled direct and mediated effects: definition, identification and bounds. Scand Stat Theory Appl. 2011;551–563. 25309023 10.1111/j.1467-9469.2010.00722.x PMC4193506

[33] Schisterman EF , Perkins NJ , Mumford SL , Ahrens KA , Mitchell EM . Collinearity and causal diagrams a lesson on the importance of model specification. Epidemiology. 2017;47–53. 27676260 10.1097/EDE.0000000000000554 PMC5131787

[34] Lever J , Krzywinski M , Atman N . Points of significance principal component analysis. Nat Methods. 2017;641–642.

[35] Frysz M , Howe L , Tobias J , Paternoster L . Using SITAR (superimposition by translation and rotation) to estimate age at peak height velocity in Avon Longitudinal Study of Parents and Children [version 1; referees: 2 approved]. Wellcome Open Res. 2018;90. 30345378 10.12688/wellcomeopenres.14708.1 PMC6171559

[36] Labrecque JA , Swanson SA . Interpretation and potential biases of Mendelian randomization estimates with time‐varying exposures. Am J Epidemiol. 2019;231–238. 30239571 10.1093/aje/kwy204

[37] Xu L , Borges MC , Hemani G , Lawlor DA . The role of glycaemic and lipid risk factors in mediating the effect of BMI on coronary heart disease: a two‐step, two‐sample Mendelian randomisation study. Diabetologia. 2017;2210–2220. 28889241 10.1007/s00125-017-4396-y PMC6342872

[38] Cheng S , Rhee EP , Larson MG , Lewis GD , McCabe EL , Shen D , Palma MJ , Roberts LD , Dejam A , Souza AL , et al. Metabolite profiling identifies pathways associated with metabolic risk in humans. Circulation. 2012;2222–2231. 22496159 10.1161/CIRCULATIONAHA.111.067827 PMC3376658

[39] Wurtz P , Havulinna AS , Soininen P , Tynkkynen T , Prieto‐Merino D , Tillin T , Ghorbani A , Artati A , Wang Q , Tiainen M , et al. Metabolite profiling and cardiovascular event risk: a prospective study of 3 population‐based cohorts. Circulation. 2015;774–785. 10.1161/CIRCULATIONAHA.114.013116 PMC4351161 25573147

[40] Wang TJ , Larson MG , Vasan RS , Cheng S , Rhee EP , McCabe E , Lewis GD , Fox CS , Jacques PF , Fernandez C , et al. Metabolite profiles and the risk of developing diabetes. Nat Med. 2011;448–453. 21423183 10.1038/nm.2307 PMC3126616

[41] Tobias DK , Lawler PR , Harada PH , Demler OV , Ridker PM , Manson JE , Cheng S , Mora S . Circulating branched‐chain amino acids and incident cardiovascular disease in a prospective cohort of US women. Circ Genom Precis Med. 2018;9:e002157. 10.1161/CIRCGEN.118.002157 PMC5880282 29572205

[42] Du X , You H , Li Y , Wang Y , Hui P , Qiao B , Lu J , Zhang W , Zhou S , Zheng Y , et al. Relationships between circulating branched chain amino acid concentrations and risk of adverse cardiovascular events in patients with STEMI treated with PCI. Sci Rep. 2018;15809. 30361499 10.1038/s41598-018-34245-6 PMC6202350

[43] Audrain‐McGovern J , Benowitz NL . Cigarette smoking, nicotine, and body weight. Clin Pharmacol Ther. 2011;164–168. 21633341 10.1038/clpt.2011.105 PMC3195407

[44] Taylor AE , Richmond RC , Palviainen T , Loukola A , Wootton RE , Kaprio J , Relton CL , Davey Smith G , Munafo MR . The effect of body mass index on smoking behaviour and nicotine metabolism: a Mendelian randomization study. Hum Mol Genet. 2019;1322–1330. 30561638 10.1093/hmg/ddy434 PMC6452214

[45] Cole DA , Maxwell SE . Testing mediational models with longitudinal data: questions and tips in the use of structural equation modeling. J Abnorm Psychol. 2003;558–577. 14674869 10.1037/0021-843X.112.4.558

[46] Blakely T , McKenzie S , Carter K . Misclassification of the mediator matters when estimating indirect effects. J Epidemiol Community Health. 2013;458–466. 23386673 10.1136/jech-2012-201813

[47] Cornier MA , Despres JP , Davis N , Grossniklaus DA , Klein S , Lamarche B , Lopez‐Jimenez F , Rao G , St‐Onge MP , Towfighi A , et al. Assessing adiposity a scientific statement from the American Heart Association. Circulation. 2011;1996–2019. 10.1161/CIR.0b013e318233bc6a 21947291

[48] Soares ALG , Banda L , Amberbir A , Jaffar S , Musicha C , Price A , Nyirenda MJ , Lawlor DA , Crampin A . Sex and area differences in the association between adiposity and lipid profile in Malawi. BMJ Glob Health. 2019;9:e001542. 10.1136/bmjgh-2019-001542 PMC6747887 31565403

[49] Lawlor DA , Harbord RM , Sterne JA , Timpson N , Davey SG . Mendelian randomization: using genes as instruments for making causal inferences in epidemiology. Stat Med. 2008;1133–1163. 17886233 10.1002/sim.3034

[50] Burgess S , Daniel RM , Butterworth AS , Thompson S ; Consortium EP‐I . Network Mendelian randomization: using genetic variants as instrumental variables to investigate mediation in causal pathways. Int J Epidemiol. 2015;484–495. 25150977 10.1093/ije/dyu176 PMC4469795

