# Supplementary material for: Role of the Metabolic Profile in Mediating the Relationship Between Body Mass Index and Left Ventricular Mass in Adolescents: Analysis of a Prospective Cohort Study
Source: J Am Heart Assoc. 2020 Oct 8;9(20):e016564. doi: 10.1161/JAHA.120.016564 (PMC7763376; doi:10.1161/JAHA.120.016564)
Supplement: Supplementary file 1 — Data S1 Tables S1–S6 Figures S1–S19 [file JAH3-9-e016564-s001.pdf]

SUPPLEMENTAL MATERIAL

Data S1.

Supplemental Methods

Adiposity measures

Adiposity was measured at age 11. Waist circumference was measured to the nearest millimetre using the Harpenden anthropometric tape. Whole body DXA scans were carried out using a Lunar prodigy narrow fan beam densitometer and used to estimate total-body-less-head fat mass.

Cardiac structure measures

Cardiac structure was assessed by echocardiography in a quasi-random subset of participants in ALSPAC at the age 17 clinic. The measures used in this analysis were LVMI, LAI, LVIDD and RWT. Echocardiography was performed using a HDI 5000 ultrasound machine (Philips). All measurements were made according to the American Society of Echocardiography guidelines, where the validated equations were used to calculate LVMI and RWT (23). Average measures of LAI and LVIDD were calculated as the mean of three measurements taken.

Covariate measurements

During pregnancy, mothers of ALSPAC children were required to fill in a number of questionnaires answer questions on their age at delivery, the number of pregnancies they have had, their highest educational qualification (less than O-level, O-level, A-level or degree and above), their smoking status (ever versus never), their weight and height before pregnancy (including certainty) and household social class (based on parental occupation, education, type of neighbourhood and use of car).

Offspring covariables included sex (reported at baseline clinics) and birthweight (from birth records, obstetric data and clinic measurements). Adolescent variables considered as confounders were smoking, diet and physical activity. Smoking was self-reported by individuals at age 15 clinics. Participants were defined as a smoker if they had smoked in at least the 30 days prior to attending clinics. Although food frequency questionnaires have been carried out in ALSPAC, these were for a small subset, so although a slightly crude measure of diet fruit and vegetable intake were used to approximate healthy diets. Fruit intake was dichotomised to less than once per day or at least once per day. Vegetable intake was dichotomised to three times or less per week or at least four times per week. Physical activity was defined according to whether the individual takes part in sport with friends as reported by the individual in Focus at 15 clinics.

Age at peak height velocity was considered as a covariate in sensitivity analyses. This was estimated using Superimposition by Translation and Rotation (SITAR) mixed effects growth curve analysis. Repeated measures of height from trained fieldworkers at assessment clinics between the ages of 5 and 20, with at least one measurement for all of ages 5-<10, 10 to <15 and 15 to 20 years were used. For a full description of the methods used see *Frysz* et al (32).

Table S1. List of metabolites used in analysis.

Metabolic class	Metabolite
**Extremely large very low density lipoprotein**	Particle concentration
	Total Lipids
	Phospholipids
	Total cholesterol
	Cholesterol esters
	Free cholesterol
	Triglycerides
**Very large very low density lipoprotein**	Particle concentration
	Total Lipids
	Phospholipids
	Total cholesterol
	Cholesterol esters
	Free cholesterol
	Triglycerides
**Large very low density lipoprotein**	Particle concentration
	Total Lipids
	Phospholipids
	Total cholesterol
	Cholesterol esters
	Free cholesterol
	Triglycerides
**Medium very low density lipoprotein**	Particle concentration
	Total Lipids
	Phospholipids
	Total cholesterol
	Cholesterol esters
	Free cholesterol
	Triglycerides
**Small very low density lipoprotein**	Particle concentration
	Total Lipids
	Phospholipids
	Total cholesterol
	Cholesterol esters
	Free cholesterol
	Triglycerides
**Very small very low density lipoprotein**	Particle concentration
	Total Lipids
	Phospholipids
	Total cholesterol
	Cholesterol esters
	Free cholesterol
	Triglycerides

**Intermediate density lipoprotein**	Particle concentration
	Total Lipids
	Phospholipids
	Total cholesterol
	Cholesterol esters
	Free cholesterol
	Triglycerides
**Large low density lipoprotein**	Particle concentration
	Total Lipids
	Phospholipids
	Total cholesterol
	Cholesterol esters
	Free cholesterol
	Triglycerides
**Medium low density lipoprotein**	Particle concentration
	Total Lipids
	Phospholipids
	Total cholesterol
	Cholesterol esters
	Free cholesterol
	Triglycerides
**Small low density lipoprotein**	Particle concentration
	Total Lipids
	Phospholipids
	Total cholesterol
	Cholesterol esters
	Free cholesterol
	Triglycerides
**Very large high density lipoprotein**	Particle concentration
	Total Lipids
	Phospholipids
	Total cholesterol
	Cholesterol esters
	Free cholesterol
	Triglycerides
**Large high density lipoprotein**	Particle concentration
	Total Lipids
	Phospholipids
	Total cholesterol
	Cholesterol esters
	Free cholesterol
	Triglycerides
**Medium high density lipoprotein**	Particle concentration
	Total Lipids
	Phospholipids
	Total cholesterol

	Cholesterol esters
	Free cholesterol
	Triglycerides
**Small high density lipoprotein**	Particle concentration
	Total Lipids
	Phospholipids
	Total cholesterol
	Cholesterol esters
	Free cholesterol
	Triglycerides
**Lipoprotein particle size**	Very large density lipoprotein particle size
	Large density lipoprotein particle size
	High density lipoprotein particle size
**Cholesterol**	Total Cholesterol
	Very low density lipoprotein cholesterol
	Remnant cholesterol
	Low density lipoprotein cholesterol
	High density lipoprotein cholesterol
	Low density lipoprotein cholesterol 3
	Low density lipoprotein cholesterol 2
	Esterified cholesterol
	Free cholesterol
**Glycerides and phospholipids**	Triglycerides
	Low density lipoprotein triglycerides
	High density lipoprotein triglycerides
	Diacylglycerol
	Phosphoglycerides
	Phosphatidylcholine and other cholines
	Cholines
**Apolipoprotein**	Apolipoprotein A-I
	Apolipoprotein B
	Apolipoprotein B/A-I
**Fatty acids**	Total fatty acids
	Fatty acid chain length
	Degree of unsaturation
	Docosahexaenoic acid
	Linoleic acid
	n-3 fatty acids
	n-6 fatty acids
	Polyunsaturated fatty acids
	Monounsaturated fatty acids
**Fatty acid ratios**	Docosahexaenoic acid (%)
	Linoleic acid (%)
	Conjugated linoleic acid (%)
	n-3 fatty acids (%)
	n-6 fatty acids (%)

	Monounsaturated fatty acids (%)
	Saturated fatty acids (%)
**Glycolysis related metabolites**	Glucose
	Lactate
	Pyruvate
	Citrate
**Amino acids**	Alanine
	Glutamine
	Histidine
**Branched chain amino acids**	Isoleucine
	Leucine
	Valine
**Aromatic amino acids**	Phenylalanine
	Tyrosine
**Ketone Bodies**	Acetate
	Acetoacetate
	Beta-hydroxybutate
**Fluid balance**	Creatinine
	Albumin
**Inflammation**	Glycoprotein acetyls

Table S2. Table of missing data in covariables used in imputations models.

**Maternal covariates**	N observations	N outliers recoded as missing	N missing (including recoded as missing)
Maternal Age	954	50	50
Maternal Parity	933	7	71
Mother's highest qualification	935		69
Maternal pre-pregnancy BMI	873	63	131
Maternal pre-pregnancy height (inches)	917	19	87
Household social class	910		94
Maternal smoking (Ever smoker)	937	3	67
**Offspring covariates**			
Offspring birthweight (g)	941	63	63
Adolescent smoking (Smoked in last 30 days or more)	272		732
Frequency of fresh fruit consumption (less than once per day)	859		145
Frequency of fresh vegetable consumption (less than three times per week)	862		142
Takes part in sport with friends (physical activity)	1000		4

Table S3. Imputed sample study characteristics compared with complete case analysis sample and full ALSPAC sample.

	Imputed Sample (analysis sample) Mean (SD) or proportion (SE)				Eligible Sample Mean (SD) or proportion (SE)				ALSPAC All Mean (SD) or proportion (SE)			
	**N**	**All**	**Male**	**Female**	**N**	**All**	**Male**	**Female**	**N**	**All**	**Male**	**Female**
**Exposures**												
**BMI kg/m^2**	1004	19.07 (3.17)	18.72 (3.00)	19.37 (3.29)	1004	19.07 (3.17)	18.72 (3.00)	19.37 (3.29)	7106	19.11 (3.45)	18.85 (3.32)	19.38 (3.56)
**Waist Circumference (cm)**	1004	68.25 (8.85)	68.62 (9.03)	67.93 (8.69)	1004	68.25 (8.85)	68.62 (9.03)	67.93 (8.69)	7109	68.40 (9.51)	68.71 (9.73)	68.15 (9.30)
**Total body fat mass (g)**	1004	15217.25 (8397.65)	10889.04 (7246.14)	18981.27 (7469.77)	1004	15217.25 (8397.65)	10889.04 (7246.14)	18981.27 (7469.77)	5150	15378.78 (9220.42)	11431.67 (8458.80)	19065.85 (8369.86)
**Outcomes**												
**LVMI g/m$^{2.7}$**	1004	28.00 (5.87)	29.92 (5.95)	26.32 (5.27)	1004	28.00 (5.87)	29.92 (5.95)	26.32 (5.27)	2047	27.61 (5.98)	29.43 (6.28)	26.12 (5.28)
**LAI**	1004	0.00 (0.19)	-0.01 (0.24)	0.00 (0.12)	1004	0.00 (0.19)	-0.01 (0.24)	0.00 (0.12)	1916	0.00 (0.19)	-0.01 (0.24)	0.00 (0.15)
**RWT**	1004	0.38 (0.06)	0.39 (0.06)	0.37 (0.06)	1004	0.38 (0.06)	0.39 (0.06)	0.37 (0.06)	2056	0.38 (0.06)	0.38 (0.06)	0.38 (0.06)
**LVIDD Average (cm)**	1004	4.53 (0.46)	4.73 (0.49)	4.35 (0.36)	1004	4.53 (0.46)	4.73 (0.49)	4.35 (0.36)	2118	4.50 (0.44)	4.74 (0.42)	4.74 (0.42)
**Covariates (offspring)**												
**Sex (% Male)**	1004	0.30 (0.03)			1004	0.30 (0.03)			14 834	0.38 (0.01)		
**Offspring birthweight (g)**	1004	3463.90 (525.00)	3549.90 (558.76)	3389.12 (481.91)	941	3465.45 (514.14)	3555.60 (538.58)	3386.61 (478.56)	13 883	3381.51 (580.57)	3443.46 (595.93)	3339.49 (536.85)
**Adolescent smoking (% smoked in last 30 days or more)**	1004	0.54 (0.03)	0.54 (0.06)	0.54 (0.03)	272	0.55 (0.03)	0.58 (0.06)	0.54 (0.04)	1719	0.55 (0.01)	0.57 (0.02)	0.54 (0.02)
**Frequency of fresh fruit consumption (% consumed less than once per day)**	1004	0.83 (0.01)	0.85 (0.02)	0.82 (0.02)	859	0.85 (0.02)	0.86 (0.14)	0.85 (0.03)	8373	0.85 (0.01)	0.85 (0.17)	0.85 (0.01)

**Frequency of fresh vegetable consumption (% consumed less than three times per week)**	1004	0.72 (0.01)	0.71 (0.02)	0.72 (0.02)	862	0.77 (0.03)	0.71 (0.06)	0.80 (0.03)	8400	0.74 (0.01)	0.80 (0.02)	0.75 (0.02)
**Physical activity (% takes part in sport with friends)**	1004	0.64 (0.02)	0.75 (0.02)	0.55 (0.02)	1000	0.65 (0.03)	0.77 (0.05)	0.60 (0.04)	7087	0.65 (0.01)	0.77 (0.23)	0.57 (0.02)
**Covariates (maternal)**												
**Maternal Age**	1004	29.50 (4.45)	29.63 (4.30)	29.40 (4.58)	954	29.50 (4.44)	29.63 (4.28)	29.39 (4.57)	14 062	27.99 (4.97)	28.08 (5.01)	27.87 (4.93)
**Maternal Parity**	1004	0.70 (0.83)	0.68 (0.83)	0.72 (0.83)	933	0.70 (0.82)	0.68 (0.83)	0.72 (0.82)	13 111	0.84 (1.00)	0.86 (1.03)	0.83 (0.97)
**Maternal pre-pregnancy BMI**	1004	22.95 (3.57)	22.98 (3.43)	22.92 (3.70)	873	22.93 (3.54)	22.97 (3.38)	22.89 (3.68)	11 670	22.92 (3.83)	22.97 (3.84)	22.89 (3.82)
**Maternal pre-pregnancy height (inches)**	1004	64.68 (2.68)	64.78 (2.82)	64.60 (2.56)	917	64.68 (2.56)	64.78 (2.58)	64.59 (2.54)	12 370	64.56 (2.65)	64.57 (2.67)	64.53 (2.62)
**Maternal smoking (% ever smoker)**	1004	0.38 (0.02)	0.36 (0.02)	0.40 (0.02)	937	0.49 (0.03)	0.48 (0.06)	0.49 (0.04)	13 236	0.51 (0.01)	0.52 (0.02)	0.51 (0.02)
**Mother's highest qualification**	1004				935				12 323			
Less than O-level		0.15 (0.01)	0.14 (0.02)	0.17 (0.02)		0.16 (0.01)	0.13 (0.02)	0.17 (0.02)		0.30 (0.01)	0.30 (0.01)	0.30 (0.01)
O-level		0.35 (0.02)	0.34 (0.02)	0.35 (0.02)		0.35 (0.02)	0.34 (0.02)	0.35 (0.02)		0.35 (0.01)	0.35 (0.01)	0.35 (0.01)
A-level		0.29 (0.01)	0.30 (0.02)	0.27 (0.02)		0.28 (0.01)	0.31 (0.02)	0.27 (0.02)		-0.22 (0.01)	0.22 (0.01)	0.23 (0.01)
Degree or above		0.21 (0.01)	0.22 (0.02)	0.20 (0.02)		0.21 (0.01)	0.26 (0.02)	0.20 (0.02)		0.13 (0.01)	0.13 (0.01)	0.13 (0.01)
**Household social class**	1004				910				11 416			
I (highest)		0.21 (0.01)	0.24 (0.02)	0.19 (0.02)		0.21 (0.01)	0.24 (0.02)	0.19 (0.02)		0.13 (0.01)	0.14 (0.01)	0.13 (0.01)
II		0.45 (0.02)	0.47 (0.02)	0.45 (0.02)		0.45 (0.01)	0.47 (0.02)	0.45 (0.02)		0.42 (0.01)	0.41 (0.01)	0.42 (0.01)

IIINM		0.21 (0.01)	0.18 (0.02)	0.24 (0.02)		0.23 (0.01)	0.18 (0.02)	0.24 (0.02)		0.26 (0.01)	0.26 (0.01)	0.26 (0.01)
IIIM		0.08 (0.01)	0.07 (0.01)	0.08 (0.01)		0.08 (0.01)	0.07 (0.01)	0.08 (0.01)		0.13 (0.01)	0.14 (0.01)	0.13 (0.01)
IV or V (lowest)		0.04 (0.01)	0.04 (0.01)	0.04 (0.01)		0.04 (0.01)	0.04 (0.01)	0.03 (0.01)		0.06 (0.01)	0.06 (0.01)	0.06 (0.01)

Table S4. Total effects between adiposity and cardiac structure, excluding and including an interaction parameter for sex (complete case analysis).

Exposure	Outcome	Beta with no interaction parameter (95% CI)	Beta with interaction parameter (95% CI)	P value for interaction
**BMI**	LVMI	0.799 (0.567, 1.032)	0.688 (0.266, 1.11)	0.514
	LAI	-0.009 (-0.015, -0.003)	-0.015 (-0.026, -0.004)	0.216
	RWT	0.001 (-0.001, 0.004)	0.002 (-0.003, 0.006)	0.974
	LVIDD	0.035 (0.017, 0.053)	0.036 (0.003, 0.068)	0.970
**Waist Circumference**	LVMI	0.242 (0.153, 0.33)	0.191 (0.045, 0.336)	0.361
	LAI	-0.003 (-0.006, -0.001)	-0.005 (-0.008, -0.001)	0.334
	RWT	0.001 (0, 0.002)	0.001 (-0.001, 0.002)	0.733
	LVIDD	0.011 (0.005, 0.018)	0.011 (0, 0.022)	0.945
**DXA**	LVMI	2.53E-04 (1.53E-04, 3.54E-04)	1.42E-04 (-0.000024, 3.07E-04)	0.080
	LAI	-3.1E-06 (-5.6E-06, -5.5E-07)	-4E-06 (-8.2E-06, 2.11E-07)	0.578
	RWT	6.88E-07 (-4.3E-07, 1.8E-06)	7.33E-07 (-1.1E-06, 2.58E-06)	0.949
	LVIDD	1.15E-05 (4.06E-06, 1.89E-05)	5.33E-06 (-6.9E-06, 1.76E-05)	0.193

Table S5. The proportion mediated by standard cardiovascular risk factors alone, metabolites considered jointly as principal components and standard cardiovascular risk factors in addition to metabolite principle components on the association between BMI and left ventricular mass, adjusting for peak height velocity as a covariate

**Mediator**	**Female**		**Male**	
	**Indirect effect**	**Proportion mediated**	**Indirect effect**	**Proportion mediated**
**Established risk factors**	0.02 (-0.02, 0.06)	2.77 (-2.31, 7.86)	0.02 (0.00, 0.05)	2.77 (0.28, 5.26)
**Metabolites only**	0.03 (-0.04, 0.09)	3.39 (-13.72, 20.5)	0.03 (-0.02, 0.08)	3.8 (-8.65, 16.26)
**Established risk factors plus metabolites**	0.02 (0.00, 0.05)	2.77 (0.28, 5.26)	0.08 (-0.14, 0.31)	10.68 (-0.86, 22.23)

Table S6. Total effects between adiposity and cardiac structure for complete case analysis and multiply imputed data.

Exposure	Outcome	Female		Male	
		Complete Case (N = 184)	Multiply Imputed (N = 536)	Complete Case (N = 55)	Multiply Imputed (N = 437)
BMI	LVMI	0.831 (0.559, 1.102)	0.661 (0.529, 0.793)	0.620 (0.167, 1.072)	0.701 (0.525, 0.877)
	LAI	-0.007 (-0.013, -0.001)	-0.002 (-0.006, 0.001)	-0.017 (-0.034, -0.001)	-0.006 (-0.016, 0.003)
	RWT	0.001 (-0.002, 0.005)	0.001 (-0.0001, 0.003)	0.001 (-0.004, 0.005)	0.002 (2.86E-05, 0.004)
	LVIDD	0.035 (0.012, 0.057)	0.027 (0.017, 0.036)	0.036 (0.005, 0.067)	0.012 (0.027, 0.042)
Waist Circumference	LVMI	0.251 (0.139, 0.363)	0.192 (0.14, 0.243)	0.167 (0.013, 0.322)	0.188 (0.128, 0.248)
	LAI	-0.003 (-0.005, 0.0004)	-0.001 (-0.002, 0)	-0.006 (-0.011, -0.0002)	-0.003 (-0.006, 0)
	RWT	0.001 (-0.001, 0.002)	0.001 (0, 0.001)	0.0003 (-0.001, 0.002)	0.001 (0, 0.001)
	LVIDD	0.011 (0.002, 0.020)	0.009 (0.006, 0.013)	0.011 (0.0002, 0.021)	0.009 (0.004, 0.014)
DXA	LVMI	3.18E-04 (1.95E-04, 4.41E-04)	2.78E-04 (2.18E-04, 3.39E-04)	1.36E-04 (-3.50E-05, 3.06E-04)	2.09E-04 (1.35E-04, 2.84E-04)
	LAI	-2.75E-06 (-5.28E-06, -2.16E-07)	-7.30E-07 (-2.19E-06, 7.30E-07)	3.78E-06 (9.75E-06, 2.18E-06)	-1.60E-06 (-4.95E-06, 1.75E-06)
	RWT	6.74E-07 (9.17E-07, 2.27E-06)	7.47E-07 (1.93E-08, 1.47E-06)	5.18E-07 (-1.05E-06, 2.18E-06)	1.33E-06 (5.27E-07, 2.13E-06)
	LVIDD	1.55E-05 (5.71E-06, 2.53E-05)	1.38E-05 (9.64E-06, 1.81E-05)	5.35E-06 (-6.44E-06, 1.72E-05)	4.76E-06 (-1.24E-06, 1.08E-05)

Figure S1. The association between BMI and individual metabolic traits.

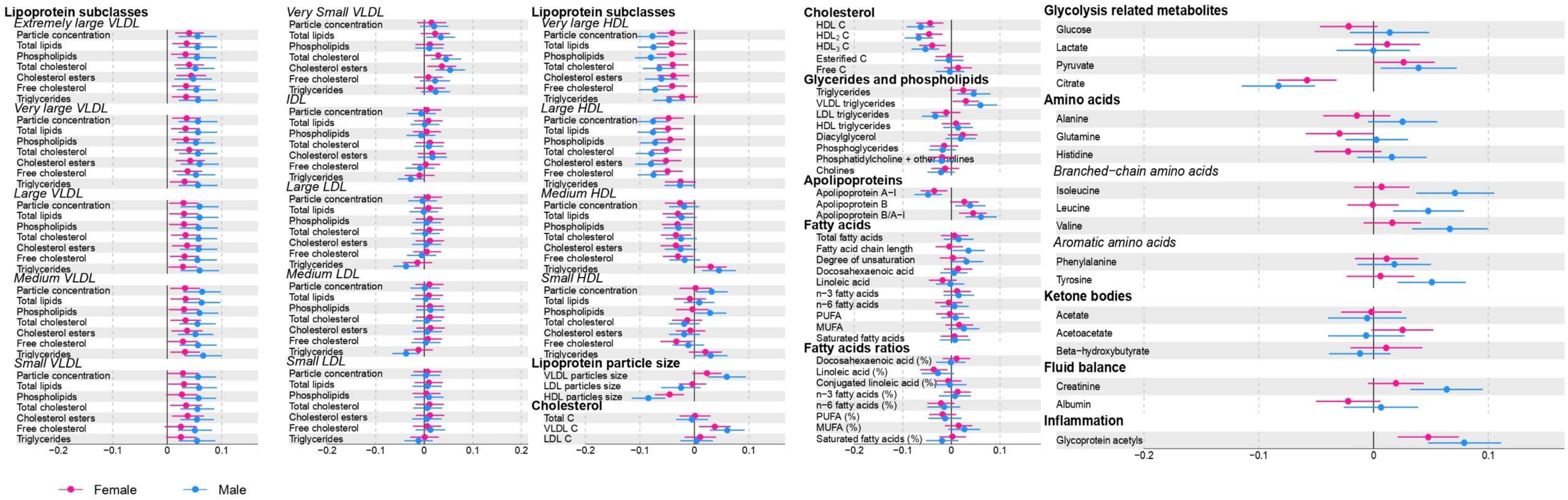

Figure S2. The association between individual metabolic traits and left ventricular mass indexed to height[2.7]

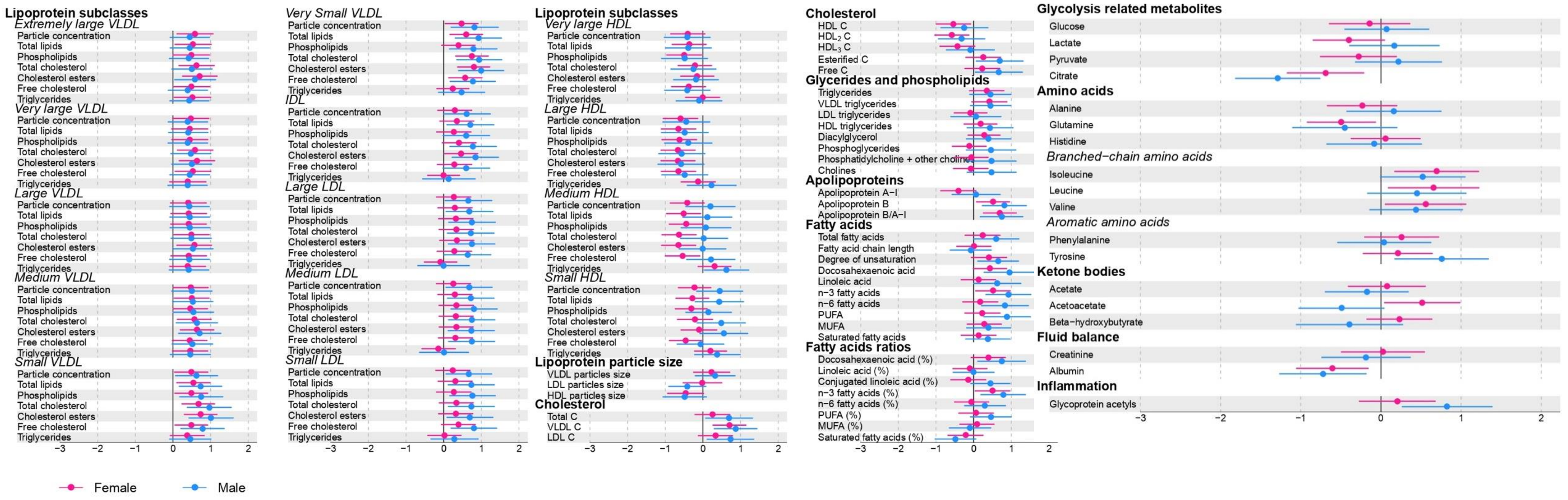

Figure S3. The association between waist circumference and individual metabolic traits.

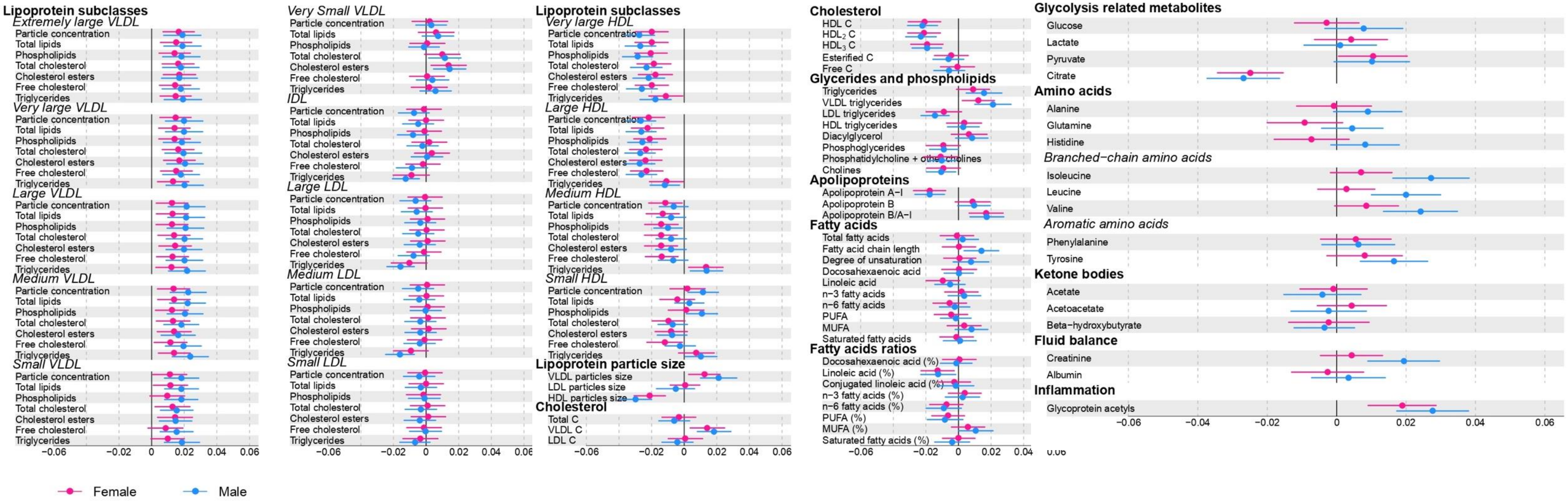

Figure S4. The association between DXA-determined fat mass and individual metabolic traits.

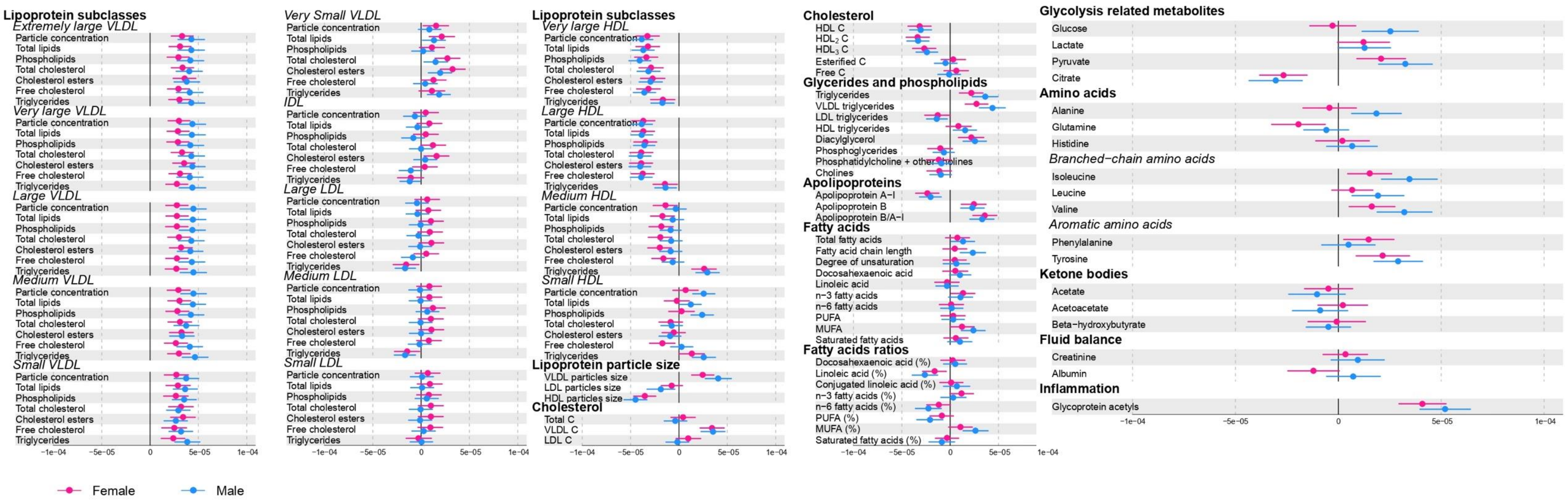

Figure S5. The association between individual metabolic traits and left atrial size indexed to height.

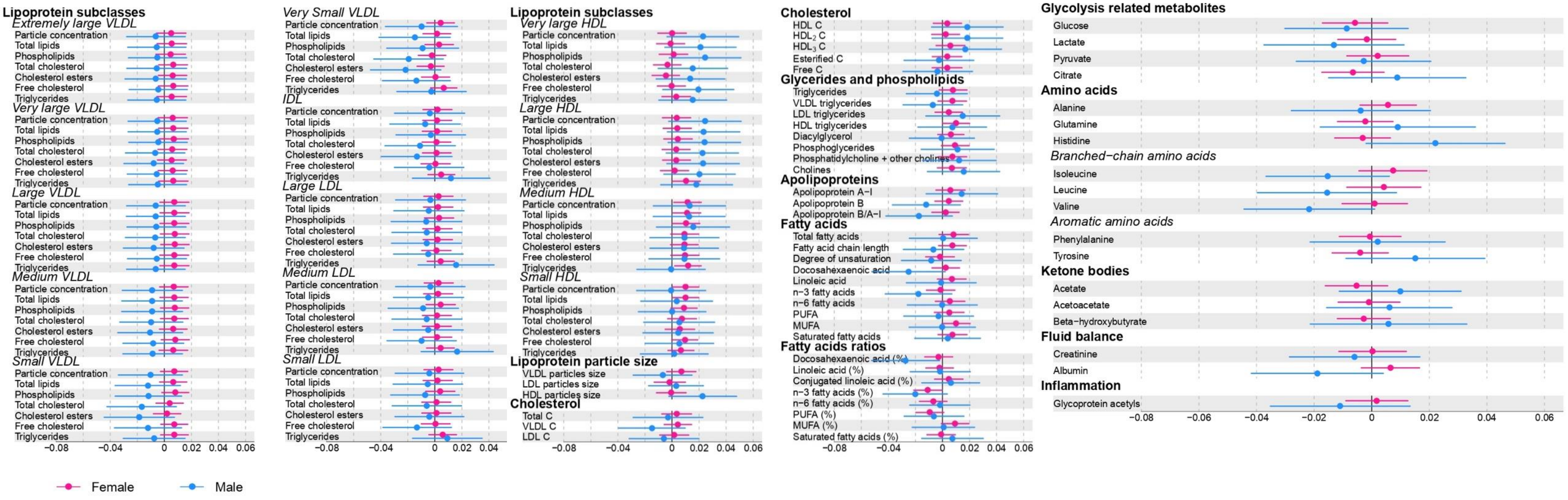

Figure S6. The association between individual metabolic traits and left ventricular internal diameter.

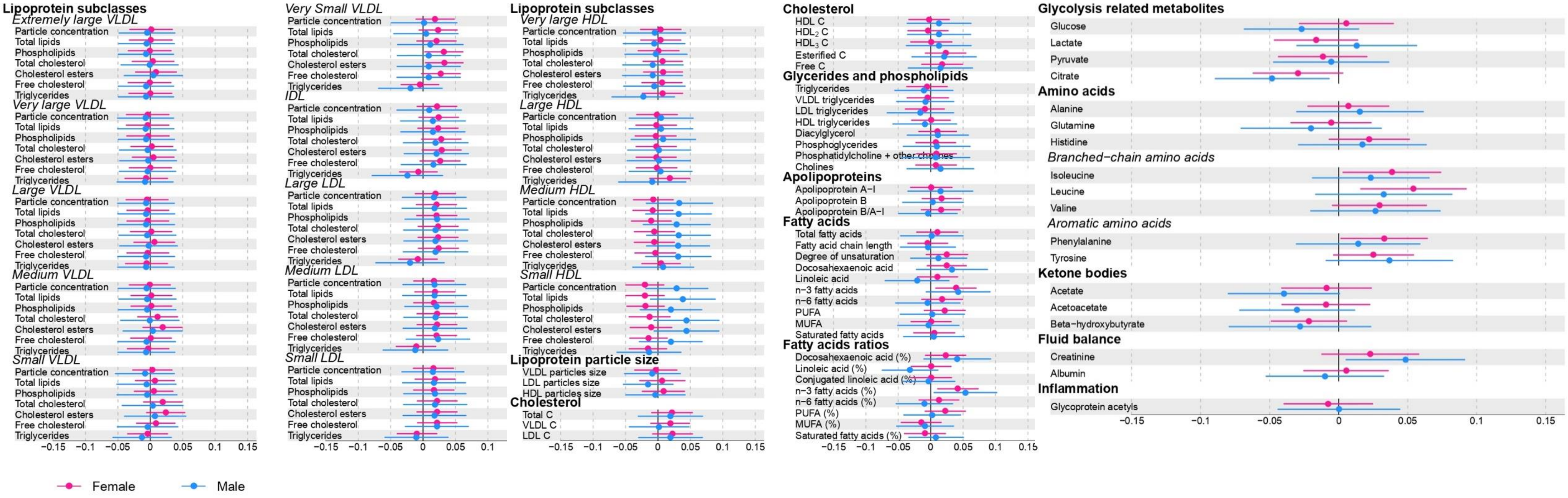

Figure S7. The association between individual metabolic traits and relative wall thickness.

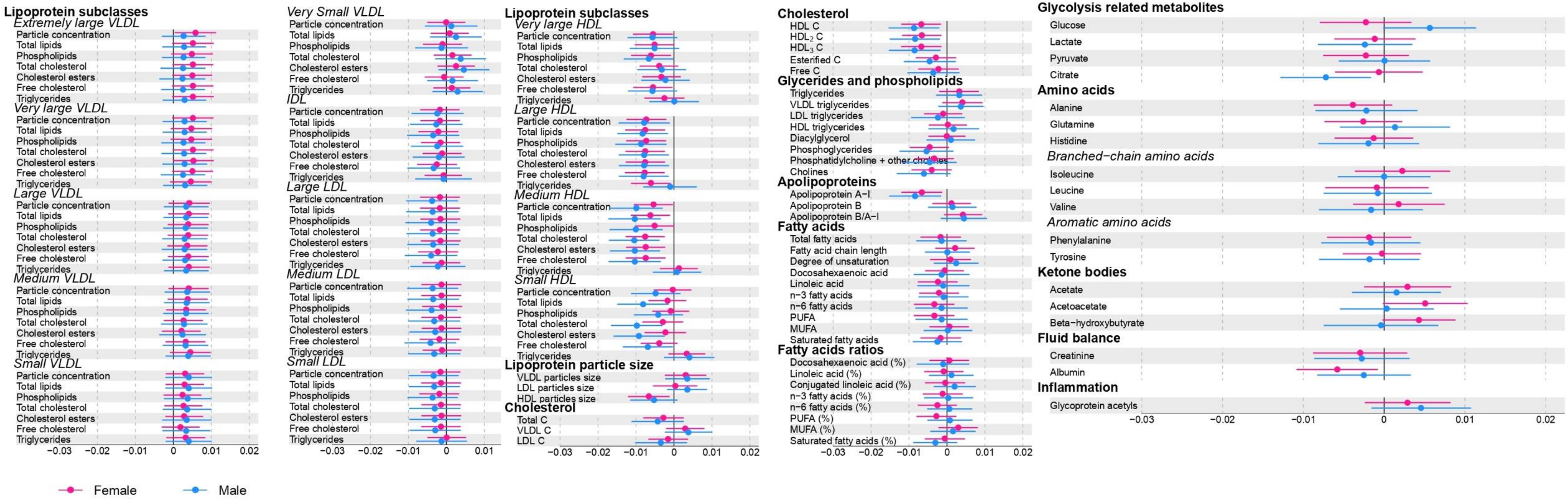

Figure S8. The indirect effect explained by each individual metabolic trait for the association of body mass index and left atrial size indexed to height

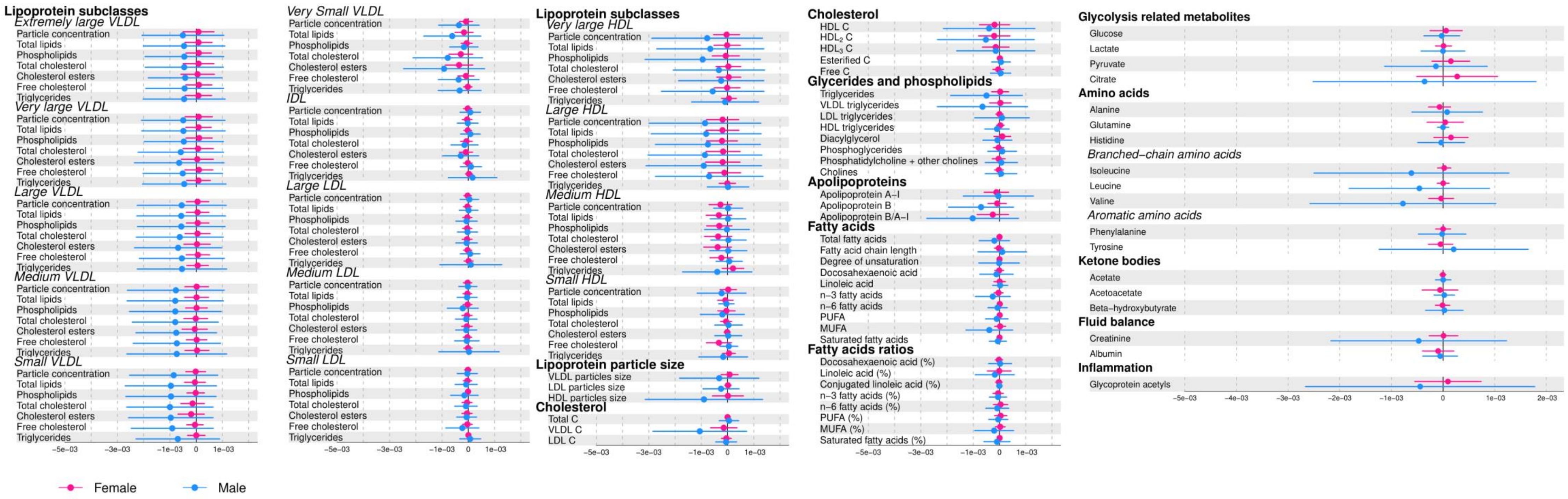

Figure S9. The indirect effect amount explained by each individual metabolic trait for the association of body mass index and left ventricular internal diameter.

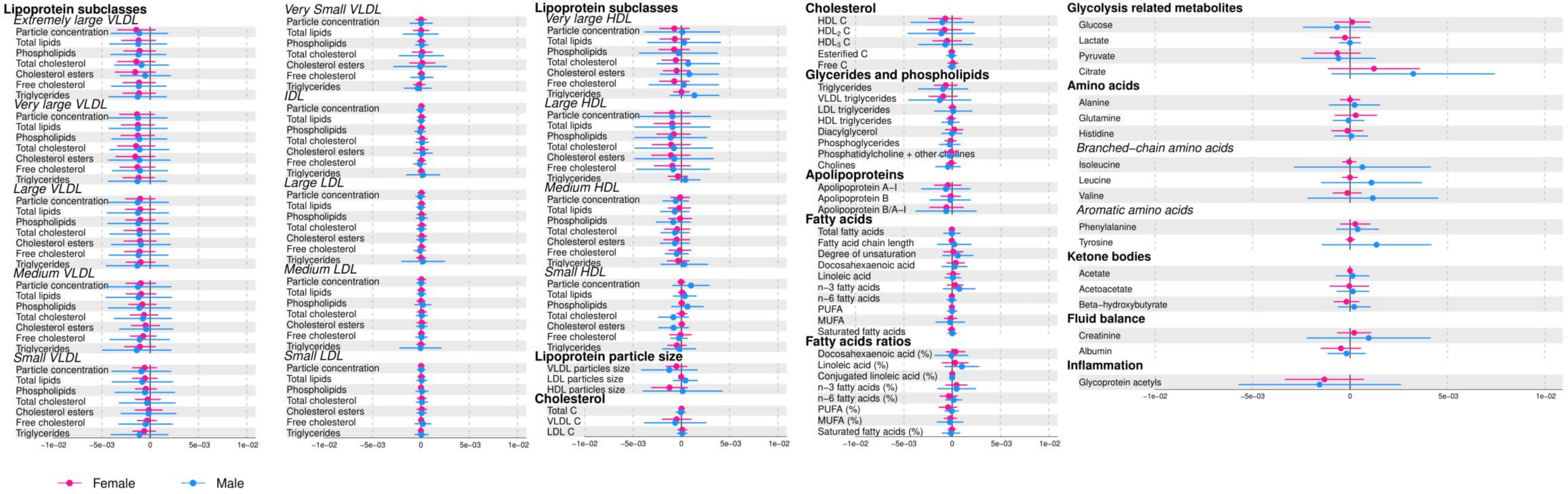

Figure S10. The indirect effect amount explained by each individual metabolic trait for the association of body mass index and relative wall thickness

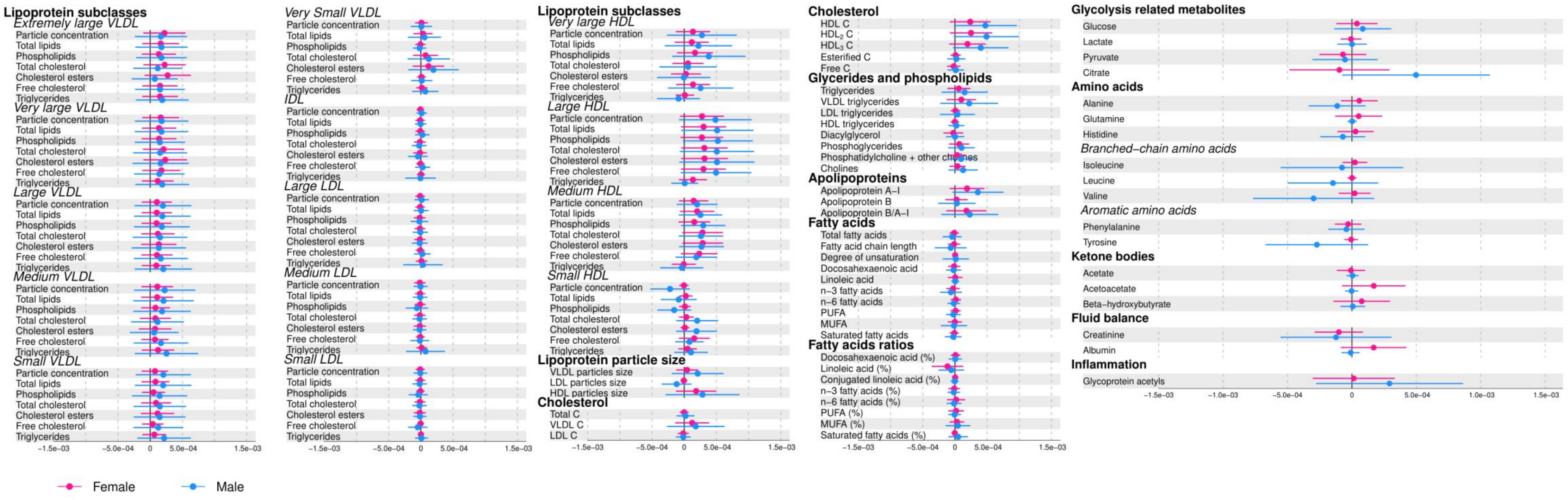

Figure S11. The indirect effect amount explained by each individual metabolic trait for the association of waist circumference and left ventricular mass indexed to height$^{2.7}$

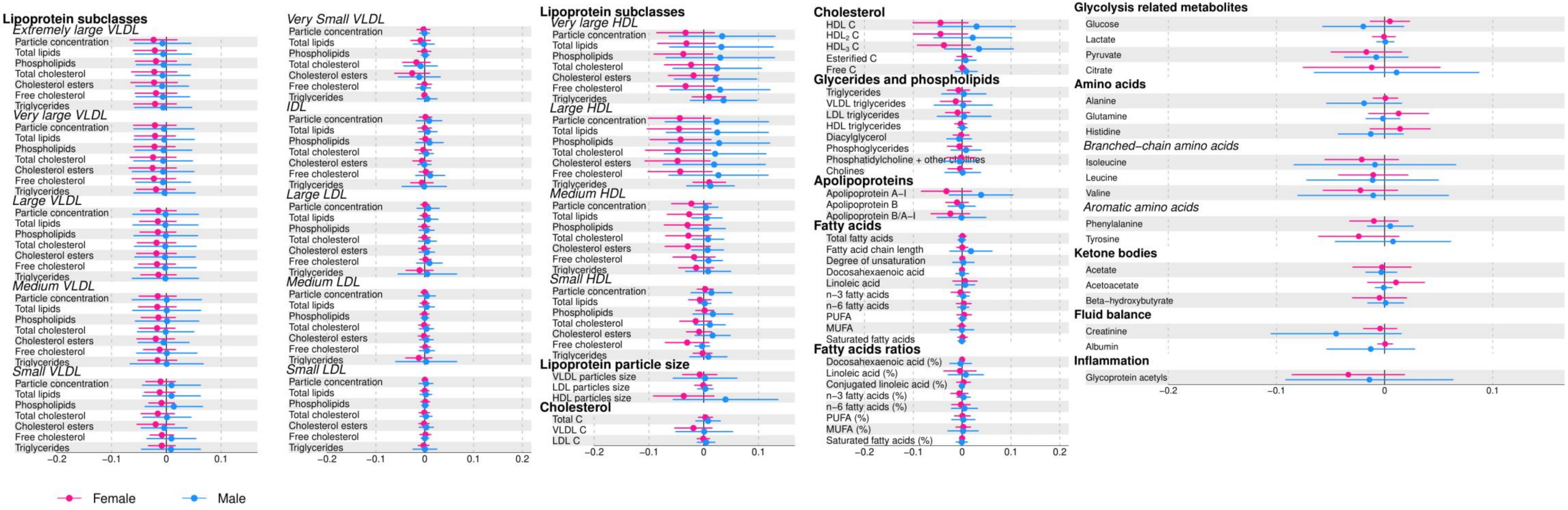

Figure S12. The indirect effect amount explained by each individual metabolic trait for the association of waist circumference and left atrial size indexed to height.

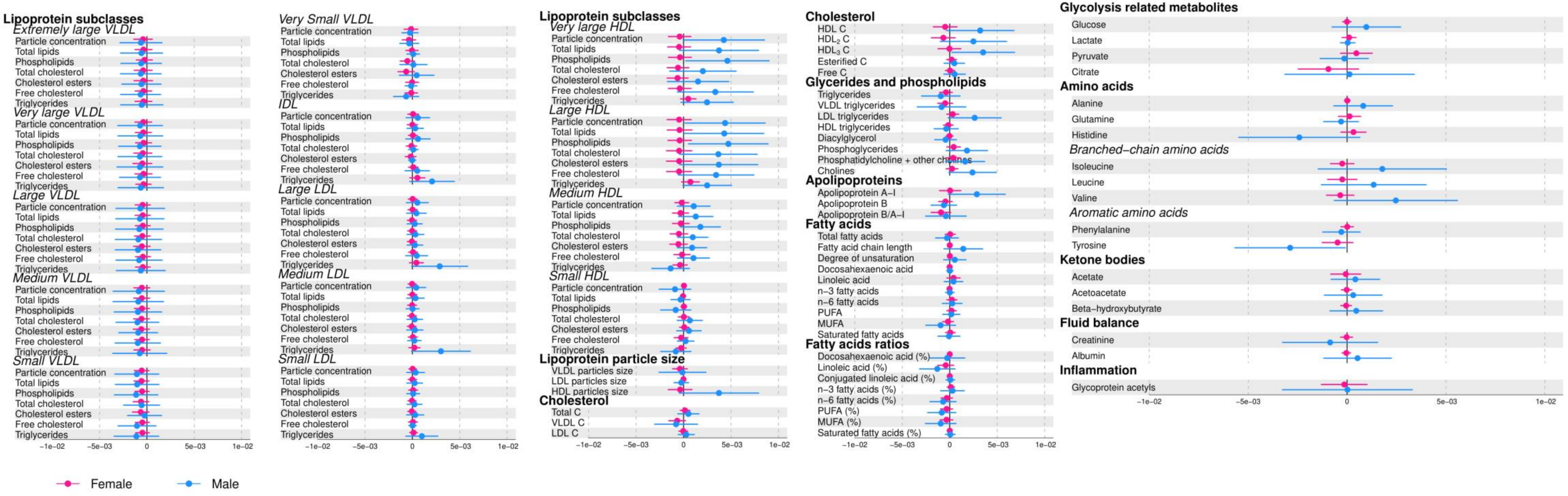

Figure S13. The indirect effect amount explained by each individual metabolic trait for the association of waist circumference and left ventricular internal diameter.

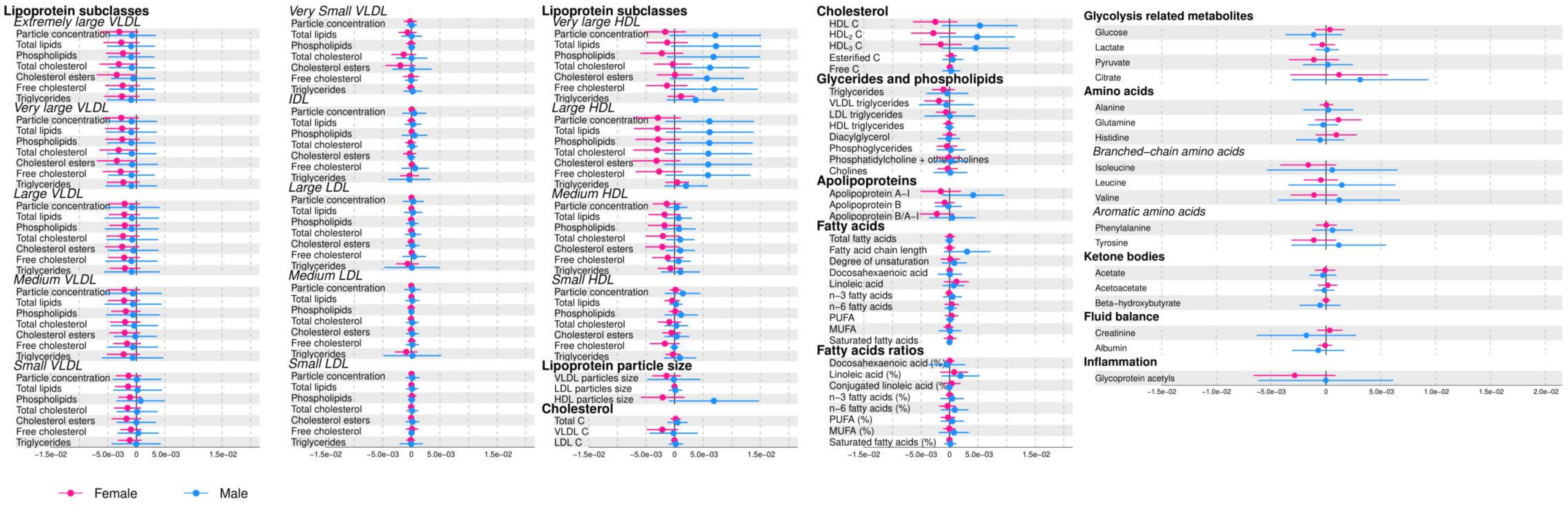

Figure S14. The indirect effect amount explained by each individual metabolic trait for the association of waist circumference and relative wall thickness

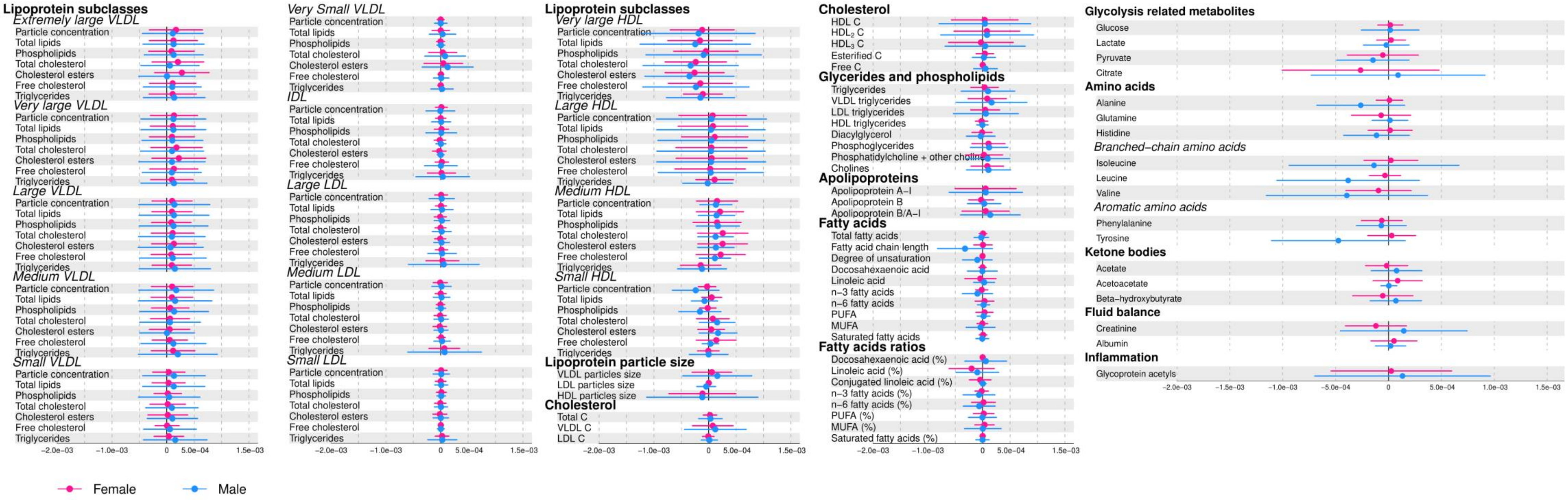

Figure S15. The indirect effect amount explained by each individual metabolic trait for the association of DXA-determined fat mass and left ventricular mass indexed to height$^{2.7}$

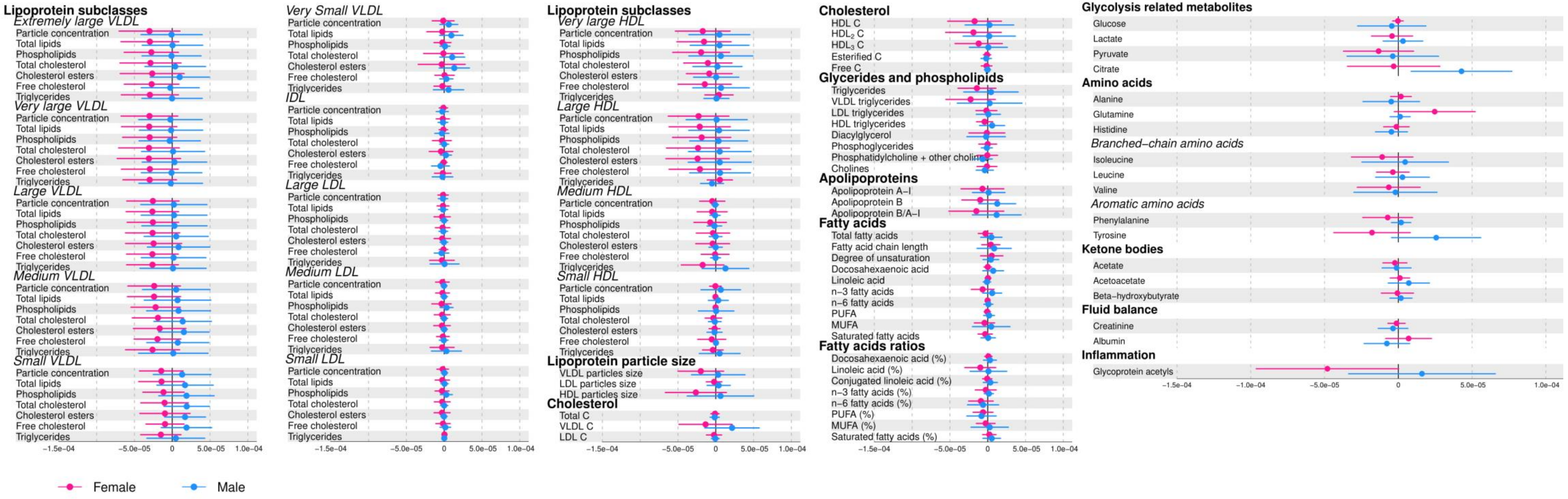

Figure S16. The indirect effect amount explained by each individual metabolic trait for the association of DXA-determined fat mass and left atrial size indexed to height

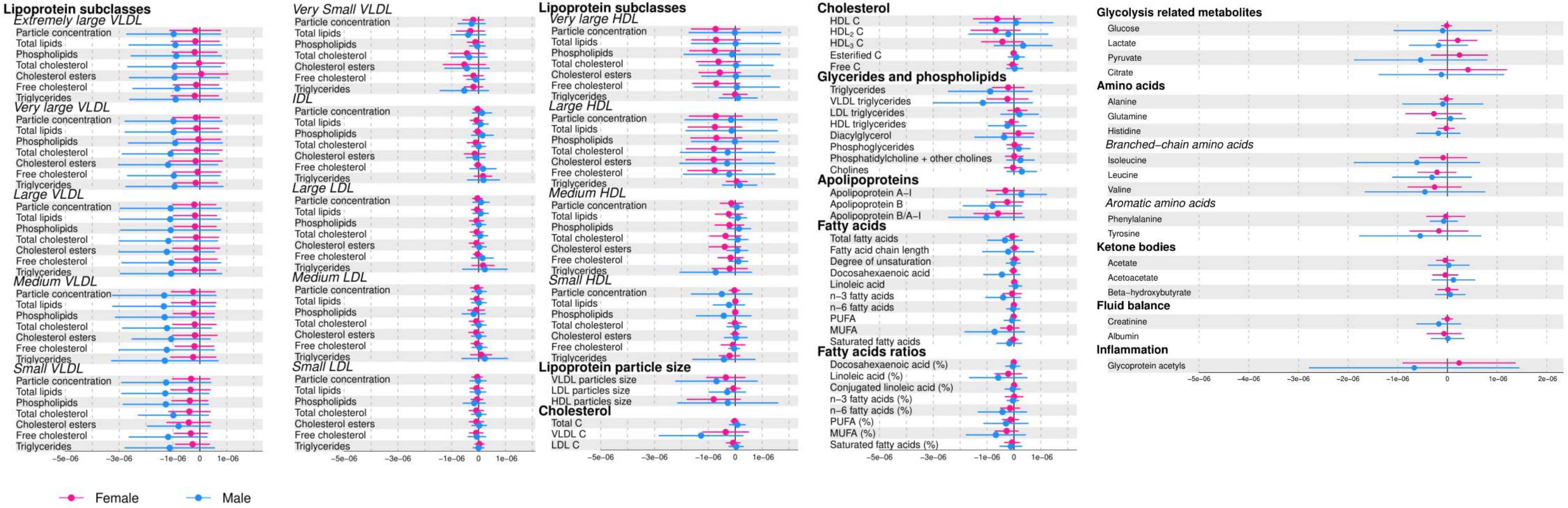

Figure S17. The indirect effect amount explained by each individual metabolic trait for the association of DXA-determined fat mass and left ventricular internal diameter

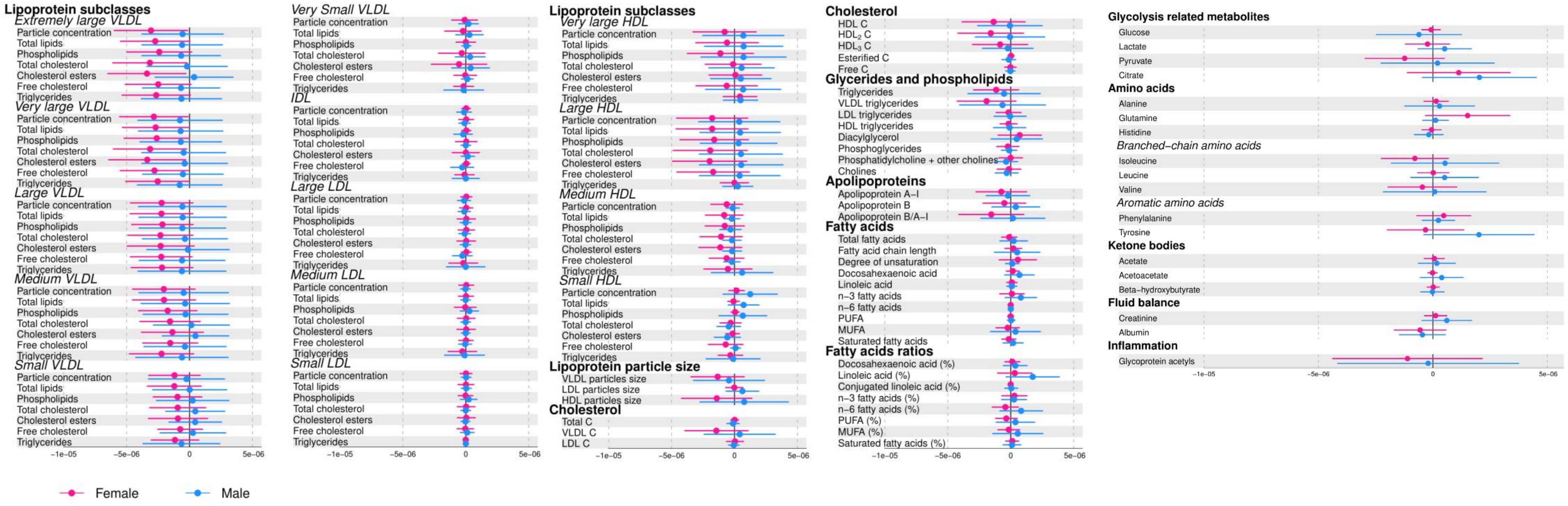

Figure S18. The indirect effect amount explained by each individual metabolic trait for the association of DXA-determined fat mass and relative wall thickness

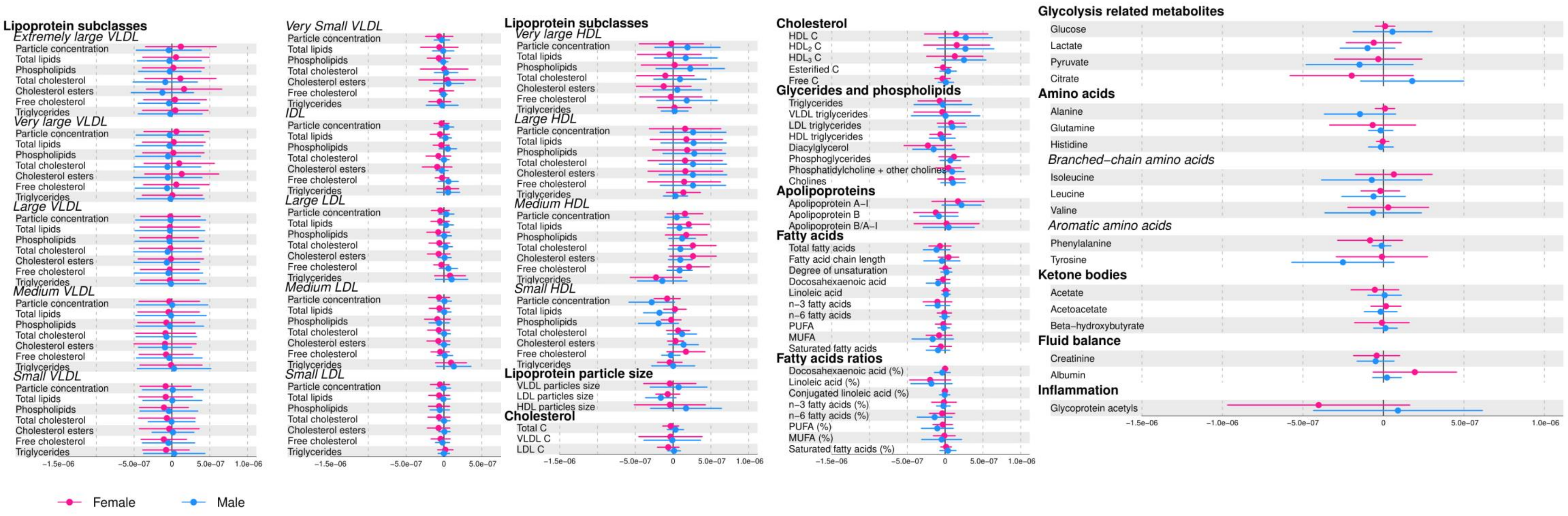

Figure S19. Forest plot showing the proportion mediated by measures of adiposity (Body mass index [BMI], waist circumference [waist] and dual x-ray absorptiometry [DXA]-determined fat mass) with cardiac structure (left atrial size indexed to height [LAI], left ventricular mass indexed to height[2.7]

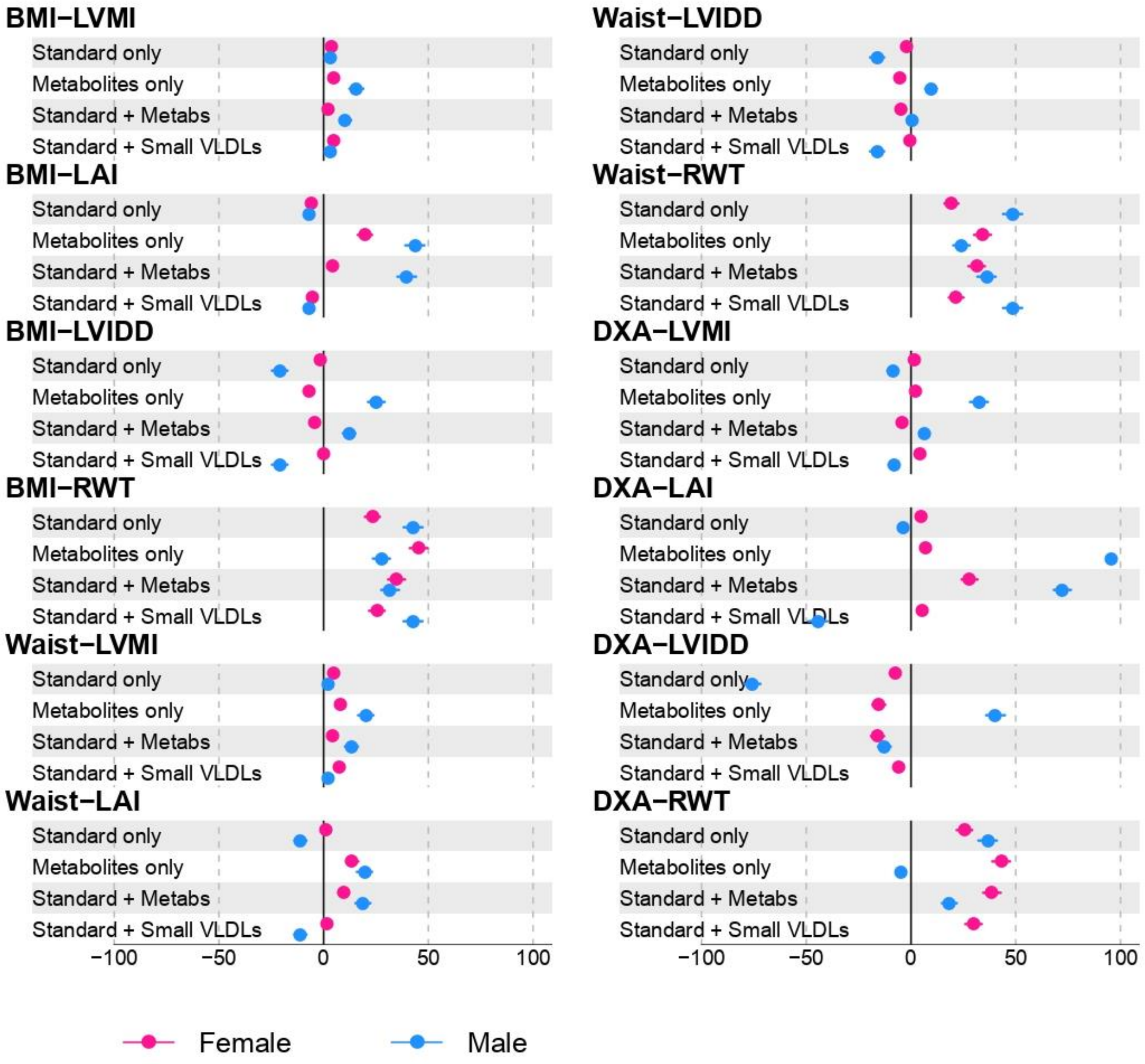

Standard mediators: systolic blood pressure, diastolic blood pressure, insulin, low density lipoprotein and glucose. Models adjusted for: Maternal age, Maternal parity, Maternal education, Maternal pre-pregnancy height, Maternal pre-pregnancy BMI, maternal smoking, household social class and adolescent birthweight. [LVMI], left ventricular internal diameter [LVIDD] and relative wall thickness [RWT]) measured using electrocardiography. Mediation was considered by i) standard risk factors ii) metabolic principal components (explaining 95% of the variation in the metabolic profile) iii) established risk factors plus metabolic PCs and iv) standard risk factors and small very low-density lipoproteins (VLDLs). Models for the effect of standard mediators plus small VLDLS in males for the association between DXA-determined fat mass and LVIDD and DXA-determined fat mass and RWT were out of the bounds of reasonable interpretation.
